# Polyphosphate synthesis is essential for phosphate and ATP homeostasis during nutrient upshift

**DOI:** 10.1073/pnas.2531128123

**Published:** 2026-06-03

**Authors:** Maria L. White, Julien Mortier, Lova Granqvist, Deike J. Omnus, Max Louski, Nick Crang, Berent Aldikacti, Valérie Migeot, Peter Chien, Marc Hennequart, Régis Hallez, Kristina Jonas

**Affiliations:** ^a^ Department of Molecular Biosciences, The Wenner-Gren Institute, Science for Life Laboratory, Stockholm University, Stockholm 10691, Sweden; ^b^ School of Engineering Sciences in Chemistry, Biotechnology and Health, Science for Life Laboratory, KTH—Royal Institute of Technology, Stockholm 10044, Sweden; ^c^ Biochemistry and Molecular Biology Department, University of Massachusetts Amherst, Amherst, MA 01003; ^d^ Molecular Physiology Research Unit, Namur Research Institute for Life Science, University of Namur, Namur 5000, Belgium; ^e^ Mass Spectrometry Facility, University of Namur, Namur 5000, Belgium; ^f^ Bacterial Cell cycle and Development, Biology of Microorganisms Research Unit, Namur Research Institute for Life Science, University of Namur, Namur 5000, Belgium

**Keywords:** PolyP, Ppk1, Pst system, nutrient adaptation, *Caulobacter crescentus*

## Abstract

Inorganic polyphosphate (polyP) is a ubiquitous molecule found across all domains of life. Although implicated in diverse cellular processes, including phosphate storage, stress responses, and pathogenicity, loss of polyP synthesis typically causes only mild growth defects. Here, we demonstrate an essential physiological role for polyP synthesis during recovery from phosphate starvation, when cells transition from phosphate-limited to phosphate-replete conditions. Using a comprehensive transposon sequencing approach in *Caulobacter crescentus*, we identify genes conferring a fitness advantage during starvation for carbon, nitrogen, or phosphate and during subsequent recovery. We find that *ppk1*, encoding the polyphosphate kinase responsible for polyP synthesis, is specifically required for recovery from phosphate starvation but dispensable for entry into starvation, a result confirmed with a *ppk1* deletion mutant. Mutations that reduce phosphate uptake via the phosphate-specific transport system suppress the requirement for *ppk1*, indicating that polyP synthesis prevents toxic accumulation of intracellular inorganic phosphate (P_i_) upon refeeding. Our findings further show that buffering intracellular P_i_ through polyP synthesis is critical for maintaining ATP homeostasis. Together, these results define a central role for polyP synthesis in regulating intracellular phosphate balance and ATP homeostasis, thereby facilitating adaptation to fluctuating nutrient conditions.

## Significance

Microorganisms often face rapid nutrient fluctuations in their environments. While much research has explored the cellular responses to starvation conditions, the mechanisms enabling recovery from starvation remain poorly studied. By identifying genetic determinants needed for nutrient adaptation in the bacterium *Caulobacter crescentus*, we reveal that the gene *ppk1* is essential for recovery from phosphate starvation. *ppk1* encodes polyphosphate kinase, which synthesizes the ubiquitous molecule polyphosphate (polyP). Our data suggest that Ppk1-dependent polyP production is critical for lowering cytoplasmic phosphate levels when external phosphorus availability rises—a condition common in aquatic environments. Cells unable to produce polyP accumulate excess ATP and remain growth-arrested. Our findings reveal an essential role of polyP synthesis and shed light on the mechanisms governing nutrient adaptation.

Most organisms must cope with periods of “feast” and “famine” in their natural environments. This is particularly the case for unicellular microorganisms that often inhabit places where nutrient conditions dramatically fluctuate. For example, in aquatic environments nutrient levels are strongly affected by seasonality and changes in microbial composition (1), but can also vary with weather conditions or due to nutrient pollution (2). Similarly, distinct host-associated environments can show drastic changes in nutrient levels (3, 4), ranging from nutrient-rich habitats in the gut to nutrient-poor habitats on the skin or within host cells. Microorganisms inhabiting and transitioning between these environments precisely adjust their growth and proliferation in response to nutrient availability to ensure their survival.

A large body of previous research has focused on the cellular processes that bacteria undergo when entering nutrient starvation. A major player in this process is the alarmone guanosine penta- and tetraphosphate, referred to as (p)ppGpp. This molecule is produced by enzymes of the RelA/SpoT superfamily upon starvation conditions and globally affects gene expression, DNA replication, and various metabolic processes by binding RNA polymerase as well as a large group of other effector proteins (5, 6). In addition to (p)ppGpp, polyphosphate (polyP) has been implicated in starvation responses in various bacteria (7, 8). PolyP is an inorganic polymer composed of phosphate monomers, whose synthesis is catalyzed by the enzyme polyphosphate kinase. It can contain over one thousand phosphate groups and forms metachromatic granules inside cells (9, 10). In contrast to (p)ppGpp, polyP is not restricted to prokaryotes, but is also produced by many other species across the three domains of life (11). PolyP has been shown to play roles in numerous different processes including phosphate storage, stress responses and virulence (7, 12). Additionally, it was shown to have chaperoning functions (13, 14). Moreover, recent research suggests that it forms biomolecular condensates that affect biophysical properties of the cell, such as chromosomal and cytoplasmic dynamics (15, 16). In different species, diverse phenotypes have been reported for mutants lacking functional polyphosphate kinase (17–23). However, in most cases the molecular basis underlying these phenotypes remains incompletely understood.

Research on bacterial starvation responses has traditionally focused on the model bacterium *Escherichia coli*. However, in particular many environmental bacteria must be able to adjust their growth and physiology in response to fluctuating nutrient levels. In addition to naturally occurring changes, these can also be caused by human activities, such as the use of fertilizer in agriculture, which can profoundly impact nitrogen and phosphate level in water bodies (2).

Among the well-studied freshwater bacteria is *Caulobacter crescentus,* an alpha-proteobacterium characterized by its dimorphic lifecycle (24, 25). This lifecycle involves the generation of distinct daughter cells by an asymmetric cell division event; a nonreplicating swarmer cell and a reproductive stalked cell. Previous research showed that in response to distinct forms of starvation, *C. crescentus* arrests the cell cycle at different stages (26, 27). Under carbon (C) and nitrogen (N) starvation *C. crescentus* delays cell differentiation from a swarmer to a stalked cell by a mechanism involving (p)ppGpp (28) and concomitantly blocks DNA replication initiation by downregulating the synthesis of the replication initiator DnaA (27, 29, 30). This results in an accumulation of nonreplicating swarmer cells under these starvation conditions (22, 27). By contrast, under phosphorus (P) starvation, *C. crescentus* arrests the cell cycle as stalked cells (27, 31, 32). Since DNA replication is blocked by a similar mechanism as under C and N starvation (27), these P-starved stalked cells are G_1_ arrested and unable to divide (27). Additionally, they exhibit strongly elongated stalks (31, 33), which are thought to improve phosphate uptake (34). While most research to date has focused on the mechanisms that allow cells to enter starvation conditions, the mechanisms facilitating the recovery from starvation remain poorly resolved. This is not only the case in *C. crescentus* but also in most other organisms.

Here, we used a randomly barcoded transposon sequencing (RB-TnSeq) approach to reveal previously uncharacterized players critical for nutrient adaptation in *C. crescentus*. We identified *ppk1*, the gene coding for polyphosphate kinase (Ppk1), to be specifically required during recovery from phosphate starvation and we show that the ability of cells to synthesize polyP is essential for resuming growth and cell cycle progression following a previous period of phosphate starvation. Our data show that polyP synthesis is critical for buffering intracellular phosphate levels and maintaining adenosine triphosphate (ATP) homeostasis when cells transition from starvation to phosphate replete conditions.

## Results

### Randomly Barcoded TnSeq Identifies Genes Potentially Important for Survival Under Starvation and Recovery Periods.

**Fig. 1.**
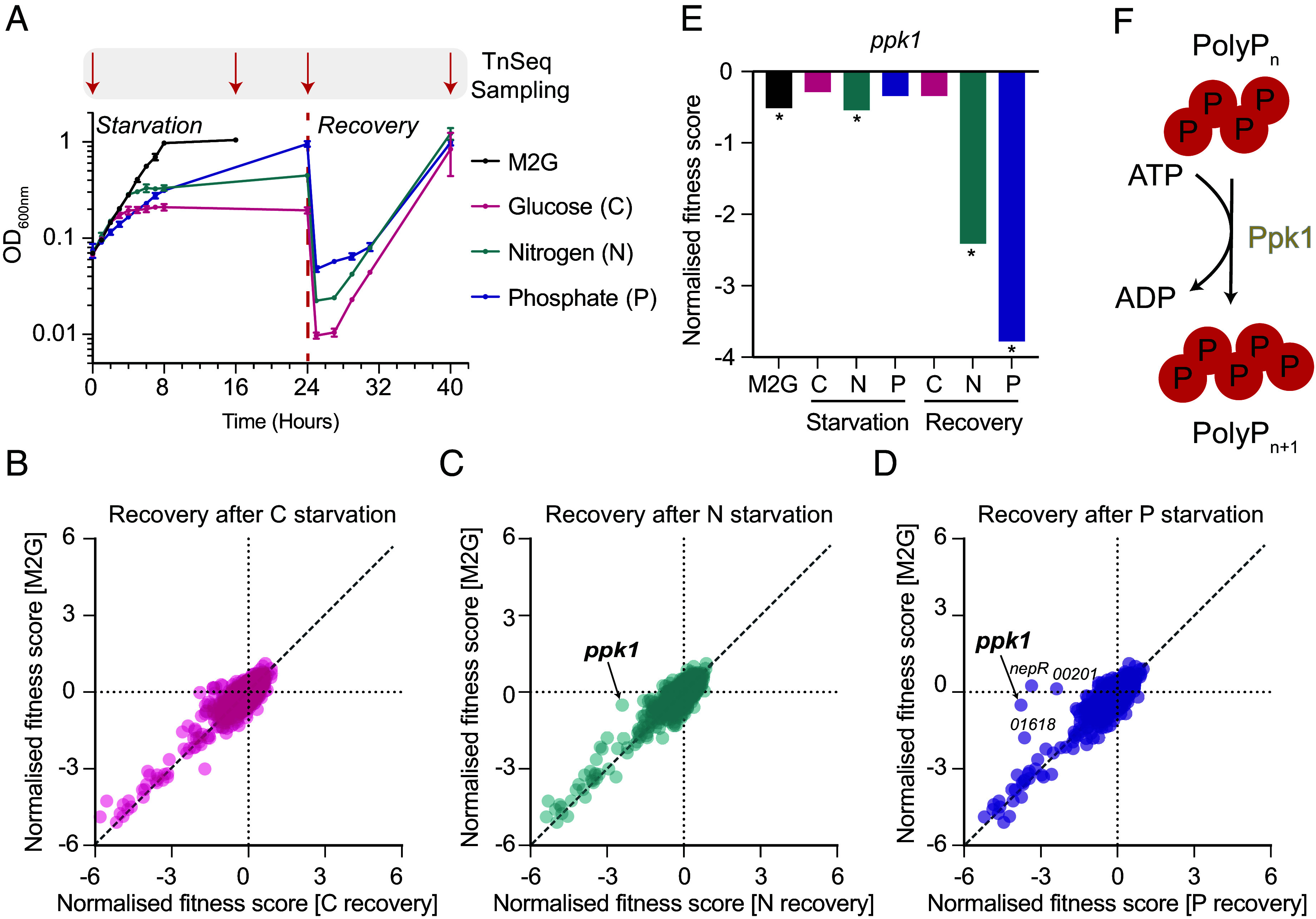
A randomly barcoded TnSeq experiment identifies genes needed for recovery from nutrient starvation in *C. crescentus.* (*A*) Growth curve of *C. crescentus* cells harboring the TnSeq library following a shift to carbon, nitrogen, or phosphate limited media for 24 h and then a shift back to nutrient replete M2G for a further 18 h. OD readings taken at 600 nm in biological triplicate. Timepoints when samples were taken for TnSeq analysis are indicated with the red arrows. (*B*) Dot plot showing normalized fitness scores for all genes after recovery from C starvation in comparison to normal growth in M2G. See also Dataset S3. (*C*) Same as in *B*, but for recovery from N starvation. (*D*) Same as in *B*, but for recovery from P starvation. (*E*) Normalized fitness scores of cells with transposon insertion in *ppk1* in all conditions tested. Asterisks indicate statistical significance, *P* < 0.05, as determined by a one-sample Student’s *t* test. (*F*) Schematic illustration of Ppk1’s activity in catalyzing the synthesis of polyP.

In order to identify genes involved in nutritional adaptation in *C. crescentus* we employed an RB-TnSeq approach (35). For this, we generated a mutant library of *C. crescentus* with randomly inserted transposons, which were identifiable by random DNA barcodes. After mapping the transposon barcodes to their genomic transposon insertion sites, we identified 44,303 transposon barcodes that reliably mapped to a single genomic location, covering 3,199 genes. The mutant library was subjected to either carbon, nitrogen, or phosphate limitation for 24 h, followed by transition to nutrient replete M2G medium for a further 18 h (Fig. 1*A*). By comparing the abundance of the barcodes between the time points before starvation (t = 0), after 24 h in the respective starvation conditions (t = 24) and after recovery from starvation (t = 42), we assessed the fitness scores of most genes under starvation and recovery conditions (Datasets S1, S2, and S3). While the fitness scores were overall relatively mildly affected by the carbon starvation and recovery conditions when compared to the fitness scores determined after growth in M2G (Fig. 1*B* and *SI Appendix*, Fig. S1), we identified several genes that showed a clearly reduced fitness score during recovery from P starvation (Fig. 1 *C* and *D*). These genes included *nepR* (encoding an anti-sigma factor), CCNA_00201 (outer membrane protein), CCNA_01618 (uracil-DNA glycosylase), and *ppk1*, which encodes polyphosphate kinase 1 in *C. crescentus* (22) (Fig. 1*D*). Transposon insertions in *ppk1* resulted in a pronounced fitness defect specifically during recovery from nitrogen and phosphate starvation (Fig. 1*E*). Given the strength of this phenotype, together with the well-established activity of Ppk1 in catalyzing the addition of the terminal phosphate group from ATP to a growing polyphosphate chain (Fig. 1*F*), and, to a lesser extent, the reverse reaction, we chose to focus on this gene.

### *ppk1* Is Required During the Phosphate Recovery Phase but not During Starvation.

**Fig. 2.**
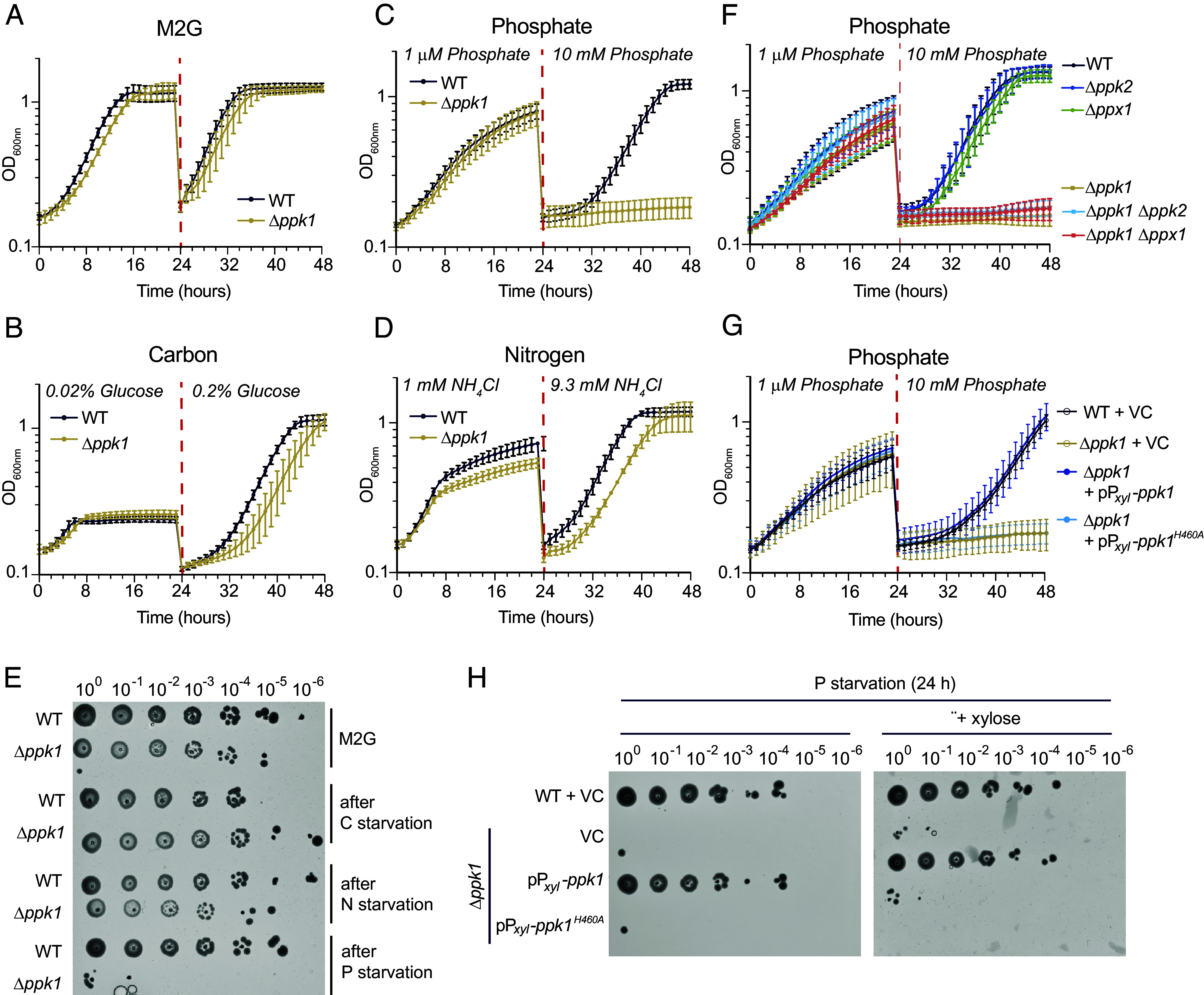
*ppk1* is essential for recovery after phosphate starvation. (*A*–*D*) Growth curves of WT *C. crescentus* and a Δ*ppk1* mutant in nutrient replete M2G (*A*), or following a shift to carbon (*B*), phosphate (*C*), or nitrogen (*D*) limitation. In all cases cells were grown for 24 h in the respective media before being diluted 1:10 into fresh nutrient replete M2G and grown for a further 24 h. Three independent experiments each in technical triplicate were carried out and the average of technical replicates plotted. (*E*) Spot assays of the WT or the Δ*ppk1* mutant following 24 h of growth in nutrient replete M2G or C, N, or P limiting media. Cultures were grown at 30 °C for 24 h before being standardized, serially diluted and plated onto nutrient replete M2G agar and incubated for 72 h at 30°. Images are representative of three independent experiments carried out in technical quadruplet. (*F*) Growth curve of the indicated strains during phosphate starvation and subsequent recovery. Cultures were grown as described previously, first in phosphate limiting media and then diluted into nutrient replete media. OD readings and replicates were collected as described for panels *A*–*D*. (*G*) Growth curve of the indicated strains containing either the vector control (VC) or a plasmid with either *ppk1* or *ppk1*^H460A^ cloned behind a xylose-inducible promoter (P*_xyl_*) during phosphate starvation and subsequent recovery. All strains were grown as in previous experiments except for glucose being swapped for 0.2 % xylose. (*H*) Spot assay of the same strains as in *G* following 24 h of phosphate limitation in either M5G or M5X media. Cultures were standardized, serially diluted and plated onto nutrient replete M2G or M2X plates before being incubated as above. Images are representative of three independent experiments carried out in technical quadruplet.

To validate our TnSeq result we used a Δ*ppk1* deletion mutant and monitored its growth, in comparison to the wild type (WT), during C, N, and P starvation and subsequent recovery (Fig. 2 *A*–*D*). Under C and N starvation and recovery conditions, the Δ*ppk1* mutant strain showed growth patterns similar to the WT. The Δ*ppk1* mutant also did not show an obvious growth phenotype during 24 h of P starvation. Moreover, live-dead staining did not indicate any loss of cell integrity in either the WT or the Δ*ppk1* mutant, even at late stages of P starvation (*SI Appendix*, Fig. S2 *A* and *B*). Importantly however, and consistent with the TnSeq data, the Δ*ppk1* mutant had a severe growth defect during recovery from phosphate starvation (Fig. 2*C*). In contrast to the WT, which resumed growth within 8 h, it was unable to restart growth after being diluted from P starvation to P rich M2G medium (Fig. 2*C*). Consistent with these growth data, we observed a similar recovery defect of the Δ*ppk1* mutant when spotting suspensions of cells that were preincubated for 24 h in P starvation media onto phosphate replete M2G agar plates (Fig. 2*E*). Notably, despite the fitness defect associated with transposon insertions in *ppk1* that we observed during recovery from nitrogen starvation in the TnSeq experiment (Fig. 1*E*), the recovery defect of the Δ*ppk1* mutant was specific to phosphate starvation, as the recovery of this strain from carbon or nitrogen starvation was not or only mildly affected by the Δ*ppk1* mutation on solid or liquid media, respectively (Fig. 2 *B, D,* and *E*).

We found that the observed phenotype during recovery from P starvation was specific to deletion of *ppk1*, as a deletion mutant of *ppk2*, encoding a second evolutionarily distinct polyphosphate kinase (36), grew as well as the WT (Fig. 2*F*). On the other hand, a Δ*ppk1* Δ*ppk2* double mutant showed a similar recovery defect as the Δ*ppk1* single mutant (Fig. 2*F*). We also investigated the phenotype of a Δ*ppx1* mutant, lacking the exopolyphosphatase Ppx1, which breaks polyphosphate (22). However, the Δ*ppx1* mutation did not notably affect cell growth during phosphate starvation and recovery (Fig. 2*F*), demonstrating that specifically Ppk1 function is needed for outgrowth from P starvation.

To determine whether loss of Ppk1’s kinase activity causes the defect in recovering from phosphate starvation we constructed a catalytically inactive variant of Ppk1 (Ppk1^H460A^), in which one of the histidine residues required for autophosphorylation (37) was substituted with an alanine residue (22). Ectopic expression of *ppk1^H460A^* in the Δ*ppk1* mutant background did not restore growth during the recovery phase after phosphate starvation, while ectopic expression of WT *ppk1* fully restored WT growth (Fig. 2 *G* and *H*). This shows that specifically the ability to synthesize polyP is required for allowing cells to resume growth upon shift from phosphate deplete to phosphate replete conditions.

### PolyP Synthesis Is Needed for Resumption of Cell Cycle Progression During Recovery from Starvation.

**Fig. 3.**
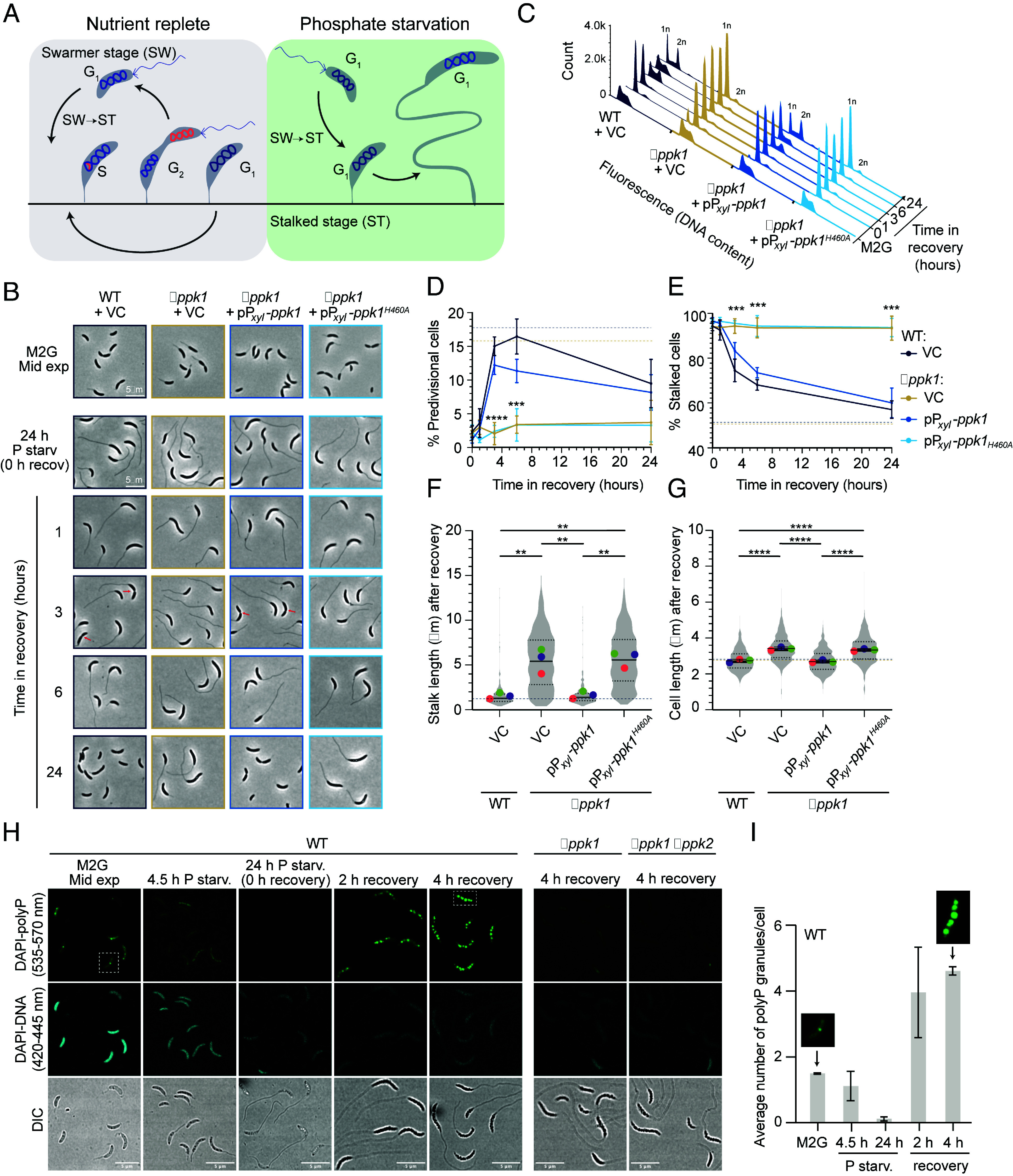
Lack of polyP synthesis prevents cells from resuming cell cycle progression during recovery from P starvation. (*A*) Schematic representation of the *C. crescentus* cell cycle under nutrient replete and phosphate starvation conditions (27). (*B*) Phase-contrast micrographs of the indicated strains. Cells were imaged live immediately after 24 h growth in phosphate limiting M5X (24 h P starv., which corresponds to 0 h recovery) and then following dilution 1:10 into phosphate replete M2X at the indicated time points. Micrographs representative of three independent experiments. (*C*) DNA content histograms as measured by flow cytometry of the indicated strains during recovery from phosphate limitation. Histograms for cells grown in M2G medium are shown for comparison. Cells were grown for 24 h in phosphate limiting M5X. Samples were analyzed immediately after shifting to nutrient replete M2X (T = 0 h) and at the indicated timepoints. 1n and 2n denote chromosome number. Histograms representative of three independent experiments. (*D* and *E*) Proportion of either predivisional cells (*D*) or non-predivisional stalked cells (*E*) at each imaged timepoint using a previously published image analysis work flow (27). Representatives of three biological replicates are shown. Asterisks denote statistical significance between the WT and the Δ*ppk1* mutant. (*F*) Violin plot of stalk lengths after 24 h recovery in nutrient replete media. The gray shaded area is representative of 375 random stalk lengths from three biological replicates. The average of each replicate is indicated. Dotted lines represent interquartile range; dashed lines represent median stalk length. Asterisks denote statistical significance for each pairwise comparison. (*G*) Violin plot of cell lengths after 24 h recovery in nutrient replete media. The gray shaded area is representative of 675 random cell lengths from three biological replicates. Replicates represented as previously described. Significance in panels *D*–*G* was determined by a One-Way ANOVA with Bonferroni correction; **P < 0.01, ***P < 0.001, ****P < 0.0001. Dotted lines in panels *D*–*G* indicate values for the WT when grown in M2G. See *SI Appendix*, Fig. S2 *A* and *B* for additional time points. (*H*) DAPI-stained polyP granules detected at 535 to 570 nm in the indicated strains. DAPI-stained DNA detected at 420 to 445 nm is shown for comparison, and DIC images visualize the cells. See also *SI Appendix*, Fig. S4, for additional data of the Δ*ppk1* and Δ*ppk1* Δ*ppk2* mutants. (*I*) Quantifications of the data for WT shown in *H*. Data points represent the average number of “green” emission maxima per cell and the error bars represent standard deviations of two independent replicates. In each replicate, the average number of polyP foci per cell was calculated from the analysis of between 119 and 334 cells.

To characterize the phenotype of the Δ*ppk1* mutant in more detail, we monitored its cell morphology and DNA replication status in comparison to the WT, or when complemented with either *ppk1* or *ppk1^H460A^*. After 24 h of phosphate starvation, all four strains had undergone the same phenotypic adaptations, as previously reported for phosphate-starved *C. crescentus* cells (Fig. 3*A*) (27, 31, 32). Essentially all cells in the population became stalked cells with significantly longer cell bodies and strongly elongated stalks and arrested DNA replication initiation as observed by an increased fraction of cells containing a single fully replicated chromosome (1n) (Fig. 3 *B* and *C*). During the recovery phase, however, the different strains showed clear *ppk1*-dependent responses. While the strains expressing functional *ppk1* were able to completely revert the phosphate starvation-induced phenotypes within 24 h after being transferred to nutrient replete conditions (Fig. 3 *B* and *C*), the Δ*ppk1* mutant and the *ppk1*^H460A^ expressing strain maintained the starvation-induced phenotype throughout the recovery period and were unable to exit the G_1_ and growth arrest despite being provided with plenty of phosphate in the growth medium (Fig. 3 *B* and *C*). Quantifications of cell types confirmed that absence of functional Ppk1 caused cells to remain as stalked cells during the recovery phase instead of progressing the cell cycle into predivisional cells, as seen for the WT and the *ppk1* complemented strain (Fig. 3 *D* and *E*). Further, after a 24 h recovery period, the Δ*ppk1* mutant and the *ppk1^H460A^* expressing strain still had strongly elongated stalks and enlarged cell bodies (Fig. 3 *F* and *G* and *SI Appendix*, Fig. S3). Together, these results show that polyP synthesis is critical for cells to restart the cell cycle when shifted from phosphate starvation to nutrient replete conditions.

Given the pronounced condition-dependent growth and cell cycle defects of the Δ*ppk1* mutant, we assessed Ppk1 activity during phosphate starvation and subsequent recovery by visualizing polyP granules, which can be done with DAPI staining followed by imaging at a green-yellow wavelength (535 to 570 nm) (10). Consistent with previous findings (10), WT cells grown in M2G medium contained one to two DAPI-stained polyP granules (Fig. 3 *H* and *I*). These foci progressively decreased in number during P starvation and were no longer detectable in P-starved cells after 24 h, indicating that polyP reserves are consumed over the course of starvation. Strikingly, within 2 to 4 h upon dilution into phosphate-replete medium, WT cells rapidly accumulated on average 4 to 5 intensely stained granules per cell, with some cells carrying 7 to 8 granules, consistent with a burst of polyP synthesis (Fig. 3 *H* and *I*). In stark contrast, in the Δ*ppk1* and Δ*ppk1* Δ*ppk2* mutants essentially no bright DAPI-stained foci were detectable (Fig. 3*H* and *SI Appendix*, Fig. S4). This confirms that the bright DAPI-stained foci at the green-yellow wavelength correspond to polyP granules and that Ppk1 is mainly responsible for producing them. Of note, Differential Interference Contrast (DIC) images of all three strains showed subcellular puncta after P starvation and recovery (Fig. 3*H* and *SI Appendix*, Fig. S4). These dense structures likely represent insoluble polyhydroxybutyrate (PHB) granules, which were previously observed in *C. crescentus* cells (38), especially under P starvation (39), and are thought to store excess carbon (40).

Together, our findings indicate that Ppk1 drives robust polyP synthesis during recovery from prior phosphate starvation and that this activity is critical for growth and cell cycle resumption under this condition.

### Reducing Phosphate Import Bypasses the Need for PolyP Synthesis.

**Fig. 4.**
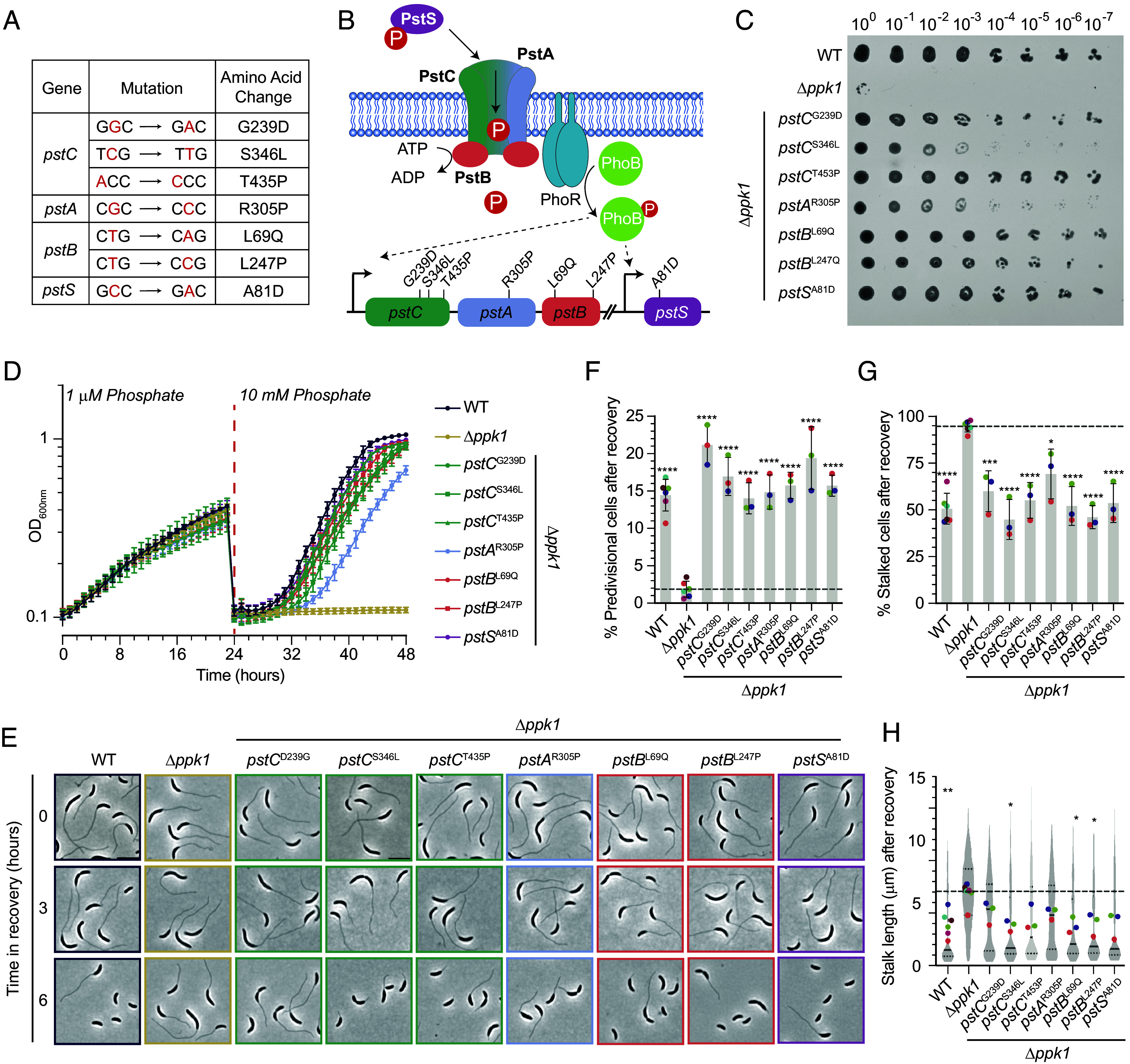
Mutations in the Pst phosphate transport system suppress the recovery defect of Δ*ppk1* cells. (*A*) List of suppressor mutations showing nucleotide changes in corresponding codons and resulting amino acid changes. (*B*) Schematic representation of the Pst system in *C. crescentus.* The location of each of the identified suppressor mutations is indicated. (*C*) Spot assay of all suppressor strains in comparison to WT *C. crescentus* and the Δ*ppk1* mutant following 24 h of phosphate limitation in M5G media. Cultures were standardized, serially diluted and plated onto nutrient replete M2G plates before being incubated for 72 h before imaging. Images are representative of three independent experiments carried out in technical quadruplet. (*D*) Growth curves of the suppressor mutants in comparison to WT *C. crescentus* and the Δ*ppk1* mutant. All strains were grown for 24 h in phosphate limiting media before being diluted 1:10 into fresh M2G. Three independent experiments each in technical triplicate were carried out and the average of technical replicates plotted. (*E*) Phase-contrast micrographs of the suppressor strains, the WT and the Δ*ppk1* mutant during recovery from phosphate starvation at the indicated time points. (Scale bar, 5 μm.) Micrographs are representatives of three biological replicates from three independent experiments. (*F*) Proportion of predivisional cells after 6 h of recovery. Data represent three biological replicates. The gray dashed line represents mean percentages for the Δ*ppk1* mutant. Statistical significance was determined and displayed by a One-Way ANOVA with Bonferroni correction, ****P < 0.0001. (*G*) Proportion of non-predivisional stalked cells after 6 h of recovery, otherwise as in *F*. See *SI Appendix*, Fig. S3 *A* and *B* for additional data of cell type proportions. (*H*) Violin plot of stalk lengths 6 h after dilution from M5G into nutrient replete media. The gray shaded area is representative of 291 random stalk lengths from three biological replicates. Dotted lines represent interquartile range; dashed lines represent median stalk length. The average of each replicate is indicated as colored circle. The gray dashed line denotes median stalk length of the Δ*ppk1* mutant. See *SI Appendix*, Fig. S3 *C* and *D* for stalk length data at additional time points as well as cell length data.

To investigate the underlying mechanism responsible for the recovery defect of the Δ*ppk1* mutant, we screened for spontaneous suppressor mutations restoring the ability of the Δ*ppk1* mutant to grow on M2G agar plates after having been exposed to either one or two subsequent 24 h periods of P starvation. This approach allowed us to isolate strains that had acquired suppressing mutations, and we subjected nine of them to whole genome resequencing to identify their mutations. Strikingly, all suppressor mutations mapped to genes encoding the phosphate-specific transport (Pst) system (Fig. 4*A*), the sole system for inorganic phosphate (P_i_) uptake in *C. crescentus*. The Pst system consists of four parts, a heterometric membrane pore complex formed by PstA and PstC, an ATPase (PstB), and a periplasmic binding protein (PstS) (Fig. 4*B*) (41, 42). We identified seven mutations in either of these four components. While the mutations *pstC^G239D^, pstC^S346L^, pstA^R305P^, pstB^L69Q^*, and *pstS^A81D^* were unique point mutations, the mutations *pstC^T453P^* and *pstB^L247P^* were isolated twice independently (Fig. 4*A*).

We characterized these seven suppressor mutants for their ability to suppress the phenotype of the Δ*ppk1* strain. We confirmed that, in contrast to the Δ*ppk1* mutant, all of them were able to grow following a 24 h P starvation period, which was the case both on agar plates (Fig. 4*C*) and in liquid medium (Fig. 4*D*). Most of the mutations restored growth to almost WT levels in the Δ*ppk1* mutant during the recovery period, whereas the mutations *pstA^R305P^* and *pstC^S346L^* (on plate) showed a more intermediate phenotype (Fig. 4 *C* and *D*). Monitoring the cell morphology and cell cycle status of the suppressor mutants during phosphate starvation and recovery showed that all of them restored WT–like cell cycle progression during the recovery period, as evident by an increased fraction of predivisional cells and a lower fraction of stalked cells (Fig. 4 *E*–*G* and *SI Appendix*, Fig. S5 *A* and *B*). Similar to the WT, but in contrast to the Δ*ppk1* mutant, the suppressor mutants also reduced stalk and cell body length during the course of recovery (Fig. 4*H* and *SI Appendix*, Fig. S5 *C* and *D*).

**Fig. 5.**
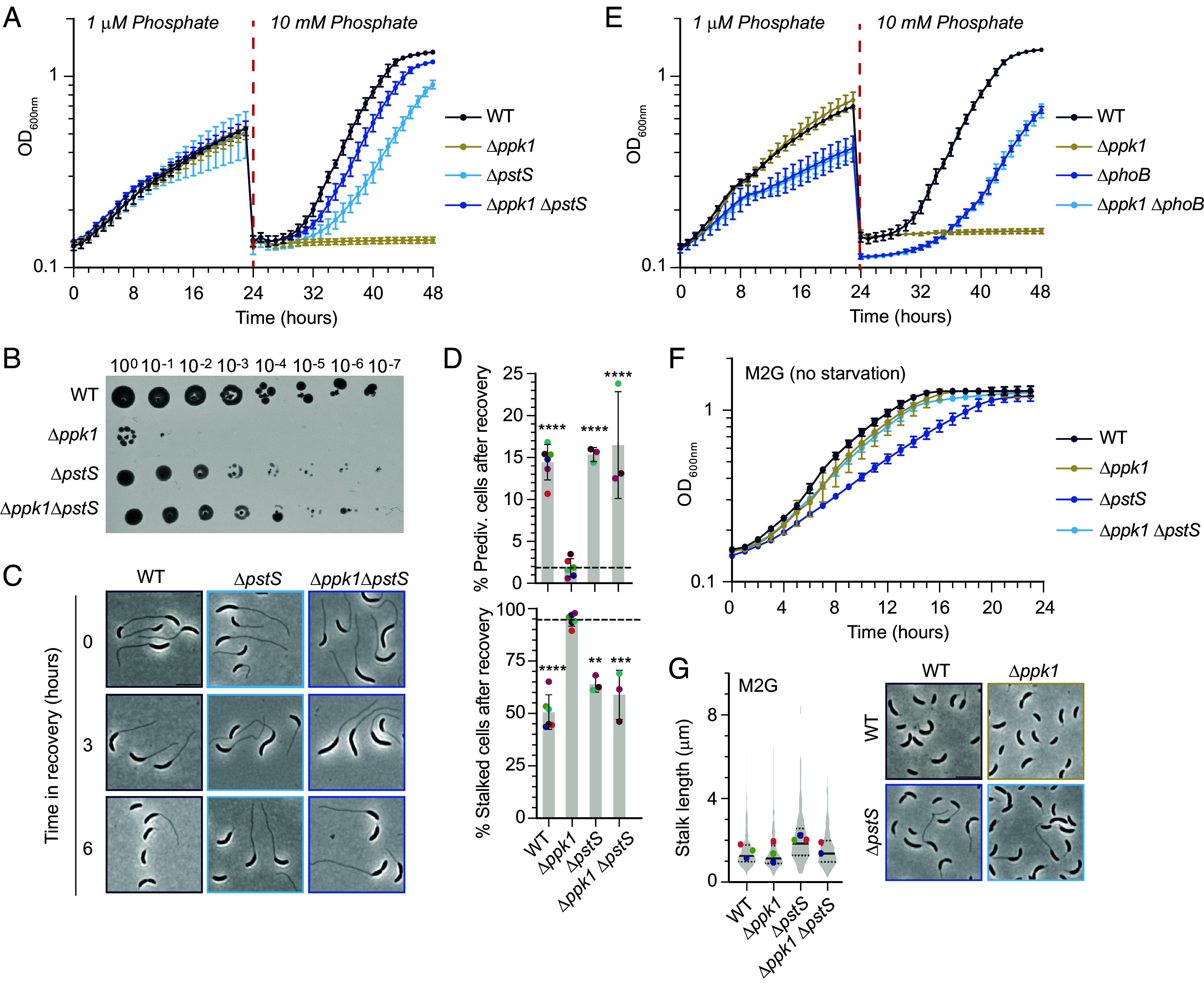
Deletions of *pstS* or *phoB* bypass the recovery defect of the Δ*ppk1* mutant. (*A*) Growth curves showing the growth phenotypes of the WT, the Δ*ppk1* mutant, the Δ*pstS* mutant and the Δ*ppk1* Δ*pstS* double mutant during phosphate starvation and subsequent recovery. All strains were grown for 24 h in phosphate limiting media before being diluted 1:10 into fresh M2G. Results are representative of three biological replicates, in technical triplicate over three independent experiments. (*B*) Spot assay of the same strains as in *A* following 24 h of phosphate limitation in M5G media. Cultures were standardized, serially diluted and plated onto nutrient replete M2G plates before being incubated for 72 h before imaging. Images are representative of three independent experiments carried out in technical quadruplet. (*C*) Phase-contrast micrographs of the indicated strains during recovery from phosphate starvation. Cells were imaged live immediately after 24 h growth in phosphate limiting M5G(T0) and then following dilution 1:10 into nutrient replete M2G, cells were imaged after 3 and 6 h. (Scale bar, 5 μm.) Micrographs representative of three biological replicates from three independent experiments. (*D*) Quantifications of proportions of predivisional cells (*Top*) and non-predivisional stalked cells (*Bottom*) after 6 h of recovery. Statistical significance was determined and displayed by a One-Way ANOVA with Bonferroni correction, ****P < 0.0001. (*E*) Growth curves showing the growth phenotypes of the WT, the Δ*ppk1* mutant, the Δ*phoB* mutant and the Δ*ppk1* Δ*phoB* double mutant during phosphate starvation and subsequent recovery. Otherwise as in *A*. (*F*) Growth curve of the strains from *A*–*D* when grown in the nonstarvation medium M2G into stationary phase. Data represent three biological replicates. (*G*) Quantifications of stalked lengths and corresponding phase-contrast micrographs for the indicated strains. Violin plots show stalk lengths with the gray shaded area representing 294 random stalk lengths from three biological replicates. Dotted lines represent interquartile ranges; dashed lines represent median stalk length. Micrographs are representative of three independent experiments. Cells were imaged live at mid-exponential phase. (Scale bar, 5 μm.)

To see if the isolated suppressor mutations were loss of function or gain of function mutations, we used a deletion mutant of *pstS*, a nonessential component of the Pst system, and tested if this mutant would also suppress the Δ*ppk1* phenotype during recovery from P starvation. Indeed, deleting *pstS* in the Δ*ppk1* background was sufficient to restore WT–like growth in liquid culture and improved growth on solid media following P starvation (Fig. 5 *A* and *B*). Furthermore, the Δ*pstS* Δ*ppk1* double mutant was able to restart the cell cycle during the recovery period, as reflected in an accumulation of predivisional cells and a decrease in stalk cell fractions (Fig. 5 *C* and *D*). This shows that the isolated suppressor mutations are loss of function mutants and are thus expected to cause a reduction of phosphate import.

The *pstCAB* and *pstS* genes are under the transcriptional control of the PhoR-PhoB two-component system, which upregulates these genes in response to phosphate starvation (31, 41, 42). Since the expression of the *pst* genes is lowered in a Δ*phoB* mutant (42), we also tested if deletion of *phoB* can rescue growth of the Δ*ppk1* mutant during recovery from phosphate starvation. As in the case of the Δ*pstS* Δ*ppk1* mutant, combining the Δ*phoB* and Δ*ppk1* mutations allowed cells to recover from starvation (Fig. 5*E*), demonstrating that lowering the expression of *pstCAB* and *pstS* by deleting *phoB* bypasses the need for *ppk1*. Notably, the Δ*phoB* Δ*ppk1* grew more slowly than the WT during P starvation and recovery, but not more slowly than the single Δ*phoB* strain (Fig. 5*E*), showing that the recovery defect of the Δ*ppk1* mutant was fully suppressed.

Finally, we also observed that the recovery defect of the Δ*ppk1* strain was largely alleviated, when diluting it in the complex medium PYE following 24 h P starvation (*SI Appendix*, Fig. S6). Because PYE contains lower P_i_ levels than M2G medium, this result indicates that lowering the extracellular P concentration of the recovery medium can also mitigate the recovery defect of the Δ*ppk1* mutant.

Together, these data show that reducing phosphate import, either by reducing the expression or the activity of the Pst transporter or by using a low P medium during recovery, bypasses the need for polyP synthesis during the recovery phase from phosphate starvation.

### The Combination of P Import and Polyphosphate Synthesis Titrates Intracellular P_i_ Levels.

Based on our data, we reasoned that polyP synthesis is needed to buffer intracellular phosphate concentrations, and more specifically to avoid excess intracellular phosphate levels when cells transition from starvation to P replete conditions.

Consistent with the idea that P import and polyP synthesis balance cytoplasmic phosphate concentrations, we found that the Δ*pstS* mutation not only suppressed the phenotype of the Δ*ppk1* strain, but that, conversely, loss of *ppk1* also improved the growth and morphological phenotypes of the Δ*pstS* strain. Due to impaired phosphate uptake via the PstCAB transporter, the Δ*pstS* mutant exhibits a growth defect as well as long stalks when grown under nonstarvation conditions in M2G and PYE medium (31) (Fig. 5*F* and *SI Appendix*, Fig. S7*A*). Furthermore, our data show that it grows more slowly than the WT during the phosphate recovery phase (Fig. 5*A*). These different defects were alleviated in the Δ*ppk1* Δ*pstS* double mutant, demonstrating that the Δ*ppk1* mutation partially compensates for the loss of Δ*pstS*, likely by increasing the available intracellular P_i_ pool (Fig. 5 *A, F,* and *G* and *SI Appendix*, Fig. S7 *A*–*C*).

These findings highlight the compensatory relationship between Ppk1 and the Pst system in titrating intracellular phosphate levels and support the idea that precise modulation of intracellular amounts of phosphate is a requirement for growth and cell cycle progression.

To seek further support for this model, we expressed *pitA^Ec^*, the phosphate transporter from *E. coli* (41), under the control of the xylose-inducible P*_xylX_* promoter at the endogenous *xylX* locus in the Δ*ppk1* and Δ*ppk2* single mutants, the Δ*ppk1* Δ*ppk2* double mutant and the WT, reasoning that PitA-dependent import of P_i_ would result in toxicity in the strains lacking Ppk1 due to their inability to convert excess intracellular P_i_ into polyP. Indeed, even leaky *pitA^Ec^* expression caused viability defects in the *ppk1*-deficient strains grown in phosphate replete conditions (M2G) (*SI Appendix*, Fig. S8). These viability defects were further exacerbated in the presence of the inducer xylose (M2GX). These results reinforce that polyphosphate synthesis is critical for eliminating the toxic effect of intracellular P_i_ accumulation.

### Absence of PolyP Synthesis Perturbs ATP Homeostasis.

**Fig. 6.**
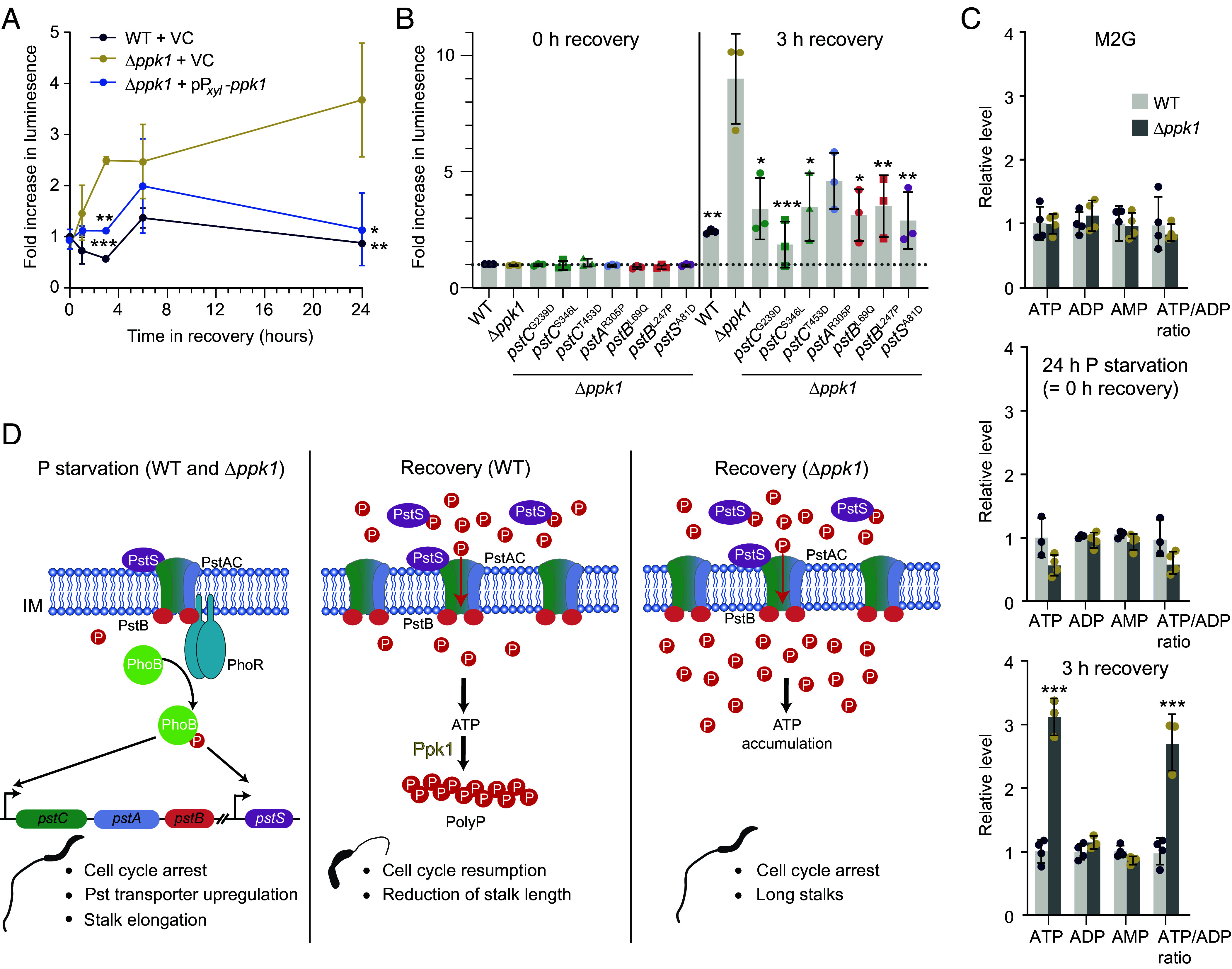
PolyP synthesis is critical for growth and ATP homeostasis during recovery from P starvation. (*A*) BacTiter-Glo assay of WT *C. crescentus* harboring the vector control (VC) and the Δ*ppk1* mutant that contains either the VC or plasmid-borne *ppk1* under the control of a xylose inducible promoter (P*_xyl_*). Cultures were grown in phosphate limiting M5XG for 24 h and samples taken immediately after dilution into fresh M2XG and 1, 3, 6, and 24 h into the recovery period. Luminescence was normalized to T = 0 h. Significance was determined by One-Way ANOVA with Bonferroni correction; *P < 0.05, **P < 0.01. Results are from three independent experiments. (*B*) BacTiter-Glo assay of the WT, the Δ*ppk1* mutant and the suppressor strains after 0 h and 3 h recovery. Results are from three biological replicates in independent experiments. Significance was determined by a One-Way ANOVA with Bonferroni correction; *P < 0.05, **P < 0.01, ***P < 0.001. (*C*) Mass spectrometry-based quantification of ATP, ADP, and AMP levels and ATP/ADP ratio in the indicated strains. In each of the conditions, nucleotide levels are shown relative to the corresponding nucleotide level in the WT. Averages from at least three independent replicates are shown with standard deviations. Statistical significance was determined by a two-way ANOVA with Tukey’s multiple comparisons; ***P < 0.001. (*D*) Model illustrating the importance of polyP synthesis during recovery from phosphate starvation. During P starvation the PhoR-PhoB two-component system upregulates the expression of *pst* genes (*Left*), leading to an upregulation of the Pst phosphate transport system. Furthermore, stalk length increases and cells arrest the cell cycle as nonreplicating stalked cells. Upon transition to P replete media, the elevated levels of phosphate transporters cause a strong influx of phosphate into the cell, which is converted into ATP. In WT cells Ppk1 buffers intracellular phosphate and ATP levels via polyP synthesis, which allows maintenance of P_i_ and ATP homeostasis (*Middle*). In Δ*ppk1* cells however, excess P_i_ and ATP cannot be converted into polyP, resulting in imbalanced homeostasis and consequently growth and cell cycle arrest (*Right*).

Previous studies showed that intracellular P_i_ and ATP levels are connected (43) and that excess ATP levels can be detrimental to cells (44–46). Based on our finding that polyP is critical for buffering cytoplasmic P_i_ levels and the notion that ATP is the substrate of Ppk1, we wondered if increased phosphate uptake upon shift to P replete conditions in the Δ*ppk1* would result in an imbalance in ATP homeostasis. To test this hypothesis, we quantified ATP levels in the WT, the Δ*ppk1* mutant, and the *ppk1* complemented strain using the BacTiter-Glo assay, which measures luminescence as a proxy for ATP levels. When grown in M2G without prior starvation all strains had similar ATP levels, with the Δ*ppk1* mutant showing mildly elevated ATP levels (*SI Appendix*, Fig. S9) and after 24 h of P starvation (t = 0 h recovery), there were no *ppk1*-dependent differences in ATP levels (Fig. 6 *A* and *B*). Interestingly however, during the first 3 h of the recovery period, the Δ*ppk1* mutant showed a significant increase in ATP levels in contrast to both the WT and the *ppk1* complemented strain and ATP levels remained high until the end of the experiment (Fig. 6*A*). Importantly, in all the suppressor mutants, ATP levels were clearly lower than in the parental Δ*ppk1* strain, with the slower growing *pstA^R305P^* suppressor mutant showing more intermediate ATP levels (Fig. 6*B*). To further corroborate the BacTiter-Glo assay findings, we quantified intracellular ATP, ADP, and AMP levels by liquid-chromatography mass spectrometry-based targeted metabolomics. While adenosine nucleotide levels were similar between the WT and the Δ*ppk1* mutant in M2G and after 24 h P starvation (Fig. 6*C*), ATP levels were significantly elevated in the Δ*ppk1* mutant after 3 h recovery, consistent with the BacTiter-Glo data. In contrast to ATP, neither ADP nor AMP was significantly affected by the Δ*ppk1* mutation; therefore, the ATP/ADP ratio is increased in the Δ*ppk1* mutant (Fig. 6*C*).

These results demonstrate that polyphosphate synthesis following excess influx of P_i_ is critical for balancing ATP levels. Furthermore, they demonstrate that the Δ*ppk1* mutant cells are still viable 24 h into the recovery period despite their growth and cell cycle arrest.

## Discussion

Inorganic polyphosphate is a ubiquitous molecule that is known to affect a wide range of cellular processes in organisms from the three domains of life. Despite its omnipresence and its association with critical cellular processes, the so far reported growth phenotypes of polyphosphate kinase mutants are often mild, which makes it difficult to assess the necessity of polyphosphate. Our study reveals an essential role of polyP synthesis during the transition from phosphate-poor to phosphate-rich environments. Our data demonstrate that the production of polyP under this condition is critical to prevent accumulation of excess intracellular monomeric phosphate and to maintain ATP homeostasis, which is necessary for growth and cell cycle progression.

Strong fluctuations in nutrient levels are a hallmark of most aquatic environments, where physiochemical parameters such as temperature and precipitation as well as human factors such as pollution with pesticides or industrial chemicals can drive periodic depletion and excess accumulation of major nutrients (47). Phosphorus is often a limiting nutrient in freshwater, however during the eutrophication process the phosphate concentration can significantly rise (47), requiring bacteria, like *C. crescentus*, inhabiting these environments to adapt to drastically changing phosphate conditions. Previous research in *C. crescentus* and other species has focused on the mechanisms allowing cells to enter growth and cell cycle arrest at the onset of nutrient starvation (22, 27, 29). However, how cells exit starvation-induced growth arrest and resume growth and proliferation when nutrients become available again remains much less studied.

Using a comprehensive TnSeq approach we uncovered *ppk1* as a gene specifically required for the recovery from P starvation. Based on our genetic and cell biological analyses of the *ppk1* mutant, we propose a model of how polyP synthesis promotes growth and cell cycle progression during the transition from P poor to P rich environments (Fig. 6*D*). During phosphate starvation, cells respond by upregulating the amounts of Pst transporters to scavenge the limiting phosphate in the environment (42). Additionally, they block DNA replication to minimize the need for phosphorus (27) and they elongate their stalks to further enhance phosphate uptake (34). When these P starved cells are shifted to P replete conditions, the high number of Pst transporters in the cell envelope will cause efficient and rapid uptake of inorganic phosphate from the outside. The imported P_i_ is expected to cause a rise in ATP concentrations (43). To counteract accumulation of excess ATP, that can cause detrimental cellular effects (44–46), Ppk1 converts ATP into polyP. While WT cells maintain in this way stable phosphate as well as ATP levels in the cell, in the Δ*ppk1* mutant P levels and ATP homeostasis are imbalanced, which results in an inability to grow and proliferate. Our data showing that cells were strictly growth and cell cycle arrested (Fig. 3) indicate that the disturbance of P_i_ and ATP homeostasis acts immediately and globally on the cell, preventing it from performing basal cellular functions.

A critical role of polyP synthesis during the transition from P poor to P rich environments is likely not only the case in *C. crescentus* and close relatives but also in diverse other organisms. In support of this idea, microalgae, yeast, and cyanobacteria are known to strongly produce polyP when resupplied with P_i_ after a period of phosphate deprivation, a phenomenon called phosphate overplus response (48–51). Indeed, there is an interest in industrial applications to utilize microbial polyphosphate production for removal of P_i_ in nutrient polluted aquatic environments (52). Furthermore, recent work in the cyanobacterium *Synechocystis sp.* PCC 6803 and the microalgae *Chlamydomonas reinhardtii* has shown that lack of a phosphate kinase results in ATP accumulation similar to what we observed here (53, 54).

In conclusion, our model of polyP synthesis serving as a system to lower toxic levels of cytoplasmic phosphate reveals insights into the physiological role of polyP. In addition to serving as a phosphate storage molecule that provides cells with phosphate during periods of P starvation, our findings demonstrate an essential role of polyP synthesis in lowering cytoplasmic monomeric P_i_ and ATP levels during the transition from low to high P environments. Future research will test the applicability of this model in other species, including other bacteria, but also eukaryotes.

## Methods and Materials

### Strain and Plasmid Construction.

All strains used in this study are derivatives of *C. crescentus* NA1000 and are listed with their construction methods in *SI Appendix*, Table S1. Details of the plasmids used in this study and how they were constructed can be found in *SI Appendix*, Tables S2 and S3. A list of all primers used for strain constructions can be found in *SI Appendix*, Table S4. For conditional expression, constructs were cloned downstream of the xylose-inducible promoter in the pRXMCS2 backbone (55). All plasmids derived from pRXMCS2 were sequenced using primer pair oMW43/oMW44. All plasmids were constructed using standard cloning procedures (PCR and Gibson Assembly) before being transformed into their target background via electroporation.

### Media and Culture Conditions.

Bacterial strains listed were routinely grown on PYE agar and subsequent liquid cultures were grown overnight in M2G media [6.1 mM Na_2_HPO_4_, 3.9 mM KH_2_PO_4_, 9.3 mM NH_4_Cl, 0.5 mM MgSO_4_, 0.5 mM CaCl_2_, 0.01 mM FeSO_4_ (EDTA-chelated; Sigma, F0518), 0.2% (w/v) glucose]. M2G/M2X agar plates were cast by mixing 3% molten Bacto agar (Difco, BD) with 2× concentrated M2G or M2X. For phosphate limiting conditions, M5G medium was used [10 mM PIPES pH 7.2, 9.3 mM NH_4_Cl, 0.5 mM MgSO_4_, 0.5 mM CaCl_2_, 0.01 mM FeSO_4_ (EDTA-chelated; Sigma, F0518), 0.2% (w/v) glucose, 1 mM NaCl, 1 mM KCl]. Phosphate was then added to the media, typically a final concentration of 1 μM was used for phosphate limiting conditions (6.1:3.9 ratio of Na_2_HPO_4_ and KH_2_PO_4_). To induce expression of genes under control of a xylose-inducible promoter, 0.2% glucose containing media was switched for 0.2% xylose containing media. 5 μg/mL kanamycin was added where required for maintenance of plasmids. All cultures were grown at 30 °C in flat-bottomed E-flasks with 200 rpm orbital shaking. Culture density was monitored using a V-1200 spectrophotometer (VWR) at 600 nm.

Nutrient limitation assays were carried out similarly to previously described (27). Briefly, a culture volume corresponding to OD_600nm_ 0.08 (when diluted to the final volume) was taken from an overnight culture. The sample was centrifuged for 10 min at 7197 × *g.* The resulting pellets were then washed in the nutrient limiting media and centrifugation was repeated. Washed cells were then resuspended in the final volume of media ready for incubation. For carbon limitation, M2G with 0.02% (w/v) glucose was used, for nitrogen limitation, M2G with 1 mM NH_4_Cl was used, phosphate limitation was carried out with M5G (1 µM phosphate) as previously described (27).

### Transposon Sequencing Experiment.

A detailed description of the TnSeq experiment can be found in *SI Appendix*. It includes descriptions of transposon mutant library construction, TnSeq sequencing library preparation, the competitive transposon mutant fitness assays and RB-TnSeq data analysis.

### Growth Curves.

One mL cultures were set-up according to previously described nutrient limiting conditions. 250 μL of each culture was then aliquoted in technical triplicate into a sterile 96-well transparent plate with flat bottom and lid. A Tecan Spark multimode microplate reader was then used for culture incubation as well as OD_600nm_ readings. Cultures were incubated at 30 °C with orbital shaking at 180 rpm. Readings were taken every hour for 24 h. To assay the recovery of cultures following starvation, all wells were diluted 1:10 into fresh nutrient-replete M2G or PYE (*SI Appendix*, Fig. S6) in a new 96-well plate and put back into the plate reader for a further 24 h. The average of the technical replicates was then plotted using GraphPad Prism (Version 10).

### Spot Assays.

Cultures were standardized to OD_600nm_ 0.1. Standardized cultures were serially diluted and 2 μL spotted onto M2G agar. Spots were left to dry before plates were inverted and incubated at 30 °C for 72 h. All plates were imaged using the Coomasie blue settings on the ChemiDoc MP (Bio-Rad). A detailed description of the spot assays to assess toxicity of *E. coli pitA* expression can be found in *SI Appendix*.

### Microscopy.

Phase contrast microscopy was carried out similar to previously described (27). Cells were spotted onto 1% (w/v) agarose pads prepared with PYE. Phase-contrast images were acquired using an ECLIPSE *Ti* inverted research microscope (Nikon) equipped with a Plan Apo λ 100× Oil Ph3 DM (1.45 NA) objective (Nikon) and a Zyla sCMOS camera (Andor). Cell type proportions and cell morphology characteristics were quantified using the workflow determined previously (27). ImageJ was used to process images acquired for figures. Statistical analysis and graphs for microscopy quantification was carried out using GraphPad Prism (Version 10).

Visualization of polyP granules was done according to a previous protocol (10). Cells were treated with 12 μg/mL DAPI that was added directly to the culture medium and incubated at room temperature in the dark for 25 min. Cells were then spotted on 1% PYE agarose pads for microscopy. Fluorescence microscopy was conducted with a Nikon Ti2 microscope equipped with an AX-R confocal scan unit, a DUVB 4ch detector unit, a transmitted light detector and a LUD-S6 laser unit. A Nikon Plan Apo λD 100× Oil OFN25 DIC N2 objective was used. The samples were scanned using simultaneous excitation and detection of three channels using 405 nm laser excitation, 2 GAASP detectors with wavelength variable filter and a transmitted light detector. DAPI-stained polyP granules were visualized by using an emission range of 535 to 570 nm (gain: 20.0). As a control, DAPI-stained DNA was imaged at an emission range of 420 to 445 nm (gain: 20.0). Transmitted light Differential Interference Contrast (DIC) imaging (gain: 30.0) was used to visualize the cells. Images were processed and polyP granules quantified with Fiji by scoring “green” emission maxima in DAPI-stained cells.

### Flow Cytometry.

Cells were sampled from growing cultures and fixed in 70% w/v ethanol before stored at 4 °C. Cells were prepared for flow cytometric quantification of cellular DNA content with SYTOX Green (Invitrogen) staining as previously described (27, 29). Cells were run on LSR Fortessa (BD Biosciences) flow cytometer (Voltages: FSC = 621, SSC = 302, FITC = 520; Threshold: FITC > 3,000), and data were plotted in FlowJo (ver. 10.7.1). Histograms were converted into isometric projects in Adobe Illustrator (Adobe) as previously described (27).

### Suppressor Screen.

To isolate potential suppressor mutants, cultures of the Δ*ppk1* mutant were standardized and grown in phosphate-limiting media as previously described. They were subsequently diluted 1:10 into 25 mL of fresh M2G. After 24 h of growth in the recovery media, cultures were serially diluted and plated onto PYE agar. After 24 h of incubation at 30 °C, single colonies were isolated on PYE agar and then screened for growth during the recovery phase. Potential suppressor mutants were identified and the genomic DNA extracted using the Wizard Genomic DNA purification kit (Promega) and then sent for whole genome sequencing (SeqCentre, Pittsburgh, PA).

In a modified screening approach, phosphate limiting cultures of the Δ*ppk1* mutant were set-up in biological triplicate as previously described. After 24 h incubation at 30 °C with shaking the cultures were diluted 1:10 into 250 μL nutrient replete M2G in a 96-well plate, each biological replicate was used to fill 32 wells. Cultures were then incubated at 30 °C with shaking for a further 24 h. The OD_600nm_ of the wells was measured using a Tecan Spark Microplate Reader. The wells with the highest OD_600nm_ were used to inoculate PYE plates. Colonies from these plates were then used to repeat the starvation and recovery passaging for enrichment of suppressor mutations. Wells that again had the highest OD_600nm_ were used to inoculate PYE plates and single colonies were isolated. 17 single colonies from six wells in total were chosen to further test for the presence of suppressor mutations.

Individual suppressor mutant candidates were then screened for growth during recovery phase that was similar to the WT control and for confirmation of the presence of the Δ*ppk1* deletion through PCR. Nine candidates were then chosen for genomic DNA extraction and whole genome resequencing as described previously.

### BacTiter-Glo Assay.

Samples were taken immediately at the respective time points/growth conditions and 100 μL of culture was standardized to OD_600nm_ 0.1. The culture was then added to 100 μL of BacTiter-Glo Reagent (Promega) in a clear bottomed black 96-well plate. For each luminescence reading, the background luminescence was controlled for with a blank containing only the culture media and the reagent. The plate was then put into a Tecan Spark Microplate reader, the plate was shaken for 5 s before being incubated at room temperature for 5 min. The luminescence was then recorded. The background luminescence was subtracted from each measurement which was then normalized to the empty vector control at T0. GraphPad Prism (Version 10) was used to plot the results and for statistical analysis.

### Metabolite Extraction and Mass Spectrometry Analysis.

*C. crescentus* cells were collected on 0.22 μm hydrophilic PVDF membranes by vacuum filtration and immediately quenched by immersing the membranes in 5 mL ice-cold lysis solvent, a methanol/acetonitrile/water solution (40:40:20, v/v/v) containing 10 µM ATP-γ-S (#NU-406, Jena Bioscience) as internal standard. Samples were vortexed vigorously and incubated at −20° C for 2 h to lyse the cells. Cell debris was removed by centrifugation at 16,000 × g for 5 min at 4 °C. The supernatants were first incubated for ≥2 h in a SpeedVac concentrator (Eppendorf Concentrator plus, Eppendorf AG, Hamburg, Germany) before being lyophilized. Dried extracts were stored at −80 °C until analysis. Dried samples were resuspended in 150 μL of methanol/acetonitrile/water (50:30:20, v/v/v) containing 10 μM ^13^C_9_,^15^N_3_-cytidine monophosphate as a technical internal standard (#650692, Merck Sigma-Aldrich). Samples were then transferred to mass-spectrometry compatible vials. Metabolite analysis was performed by LC–MS. Briefly, chromatographic separation was performed using a SeQuant Zic-pHILIC (Merck Millipore) column (5-μm particle size, polymeric, 150 mm by 2.1 mm), and metabolites were detected with a Maxis Impact I qTOF mass spectrometer (Brucker) coupled to an Ultimate 3000 HPLC (Thermo Fisher Scientific). The injection volume was 10 μL, the oven temperature was maintained at 25 °C, and the autosampler tray temperature was maintained at 4 °C. The mobile phase consisted of 12 mM ammonium carbonate in water (Eluent A) and 100% acetonitrile (Eluent B). For chromatographic separation, gradient elution started from 90% of buffer B and was programmed as follows at a flow rate of 0.3 mL/min: 0 to 17 min: 90 to 40% B; 17 to 17.2 min: 40 to 10% B; 17.2 to 22 min :10% B and then a regeneration step of 90% B from 22 to 32 min. Mass spectrometry was performed using electrospray ionization in positive mode. Source parameters were applied as follows: End plate offset, 500 V; spray voltage, 4.5 kV; nebulizer, 0.4 bar; dry gas, 4 L/min; dry temperature, 180° C; full scan range from 150 to 2,200 mass/charge ratio (m/z) at a spectra rate of 1 Hz. Data were recorded with Hystar 3.2 and analyzed with BDal compass data analysis. Peak areas for ATP, ADP, AMP, and ATPγS were extracted on the base of external standards and first normalized to the technical internal standard ^13^C_9_,^15^N3-CMP. Normalized peak areas of ATP, ADP, and AMP were then corrected with ATP-γ-S normalized peak area.

## Supplementary Material

Appendix 01 (PDF)

Dataset S01 (XLSX)

Dataset S02 (XLSX)

Dataset S03 (XLSX)

## Data Availability

All processed data can be found in the main figures and supporting information. Additional raw data and codes for the RB-TnSeq experiment are available at https://github.com/julienmortier/RB-TnSeq_starvation (56).
