# Supplementary material for: Polyphosphate synthesis is essential for phosphate and ATP homeostasis during nutrient upshift
Source: Proc Natl Acad Sci U S A. 2026 Jun 3;123(23):e2531128123. doi: 10.1073/pnas.2531128123 (PMC13250506; doi:10.1073/pnas.2531128123)
Supplement: Supplementary file 1 — Appendix 01 (PDF) [file pnas.2531128123.sapp.pdf]

Supporting Information for

Polyphosphate synthesis is essential for phosphate and ATP homeostasis during nutrient upshift

Maria L. White, Julien Mortier, Lova Granqvist, Deike J. Omnus, Max Louski, Nick Crang, Berent Aldikacti, Valérie Migeot, Peter Chien, Marc Hennequart, Régis Hallez and Kristina Jonas*

* Correspondence: Kristina Jonas, kristina.jonas@su.se

The Supporting Information includes:

Supplementary Methods
Figures S1 to S9
Tables S1 to S5
Legends for Datasets S1 to S3
SI References

Supplementary Methods

Construction of transposon mutant library

A random Tn5 transposon mutagenesis library of *C. crescentus* was created using APA 766, a diaminopimelate (DAP) auxotrophic *Escherichia coli* donor strain harboring the pKMW7 delivery vector (gifted by the Paul Hudson lab) (1). The recipient *C. crescentus* strain was grown to stationary phase overnight at 30˚C in 20 ml of PYE, while the APA 766 donor strain was grown at 37˚C in 20 ml of LB supplemented with 30 µg/ml of kanamycin and 300 µM of DAP. Next, both cultures were centrifuged (5 min, 8000 x *g*) and resuspended in 500 µl of PYE. The recipient and donor were mixed in a 10:1 ratio to a final volume of 500 ml, centrifuged (5 min, 8000 x *g*) and resuspended in 30 µl of PYE before being spotted on a PYE agar plate with 300 µM of DAP. The plate was incubated overnight at 30˚C to allow conjugation. Subsequently, the mating spot was scraped off and resuspended in 6.5 ml of PYE. The entire mixture was spread on 14 square PYE agar plates supplemented with 25 µg/ml of kanamycin and incubated for 5 days at 30˚C. Finally, the colonies were scraped from the plates and resuspended in a final volume of 70 ml of PYE containing 15 % glycerol and stored in 1 ml aliquots at -80˚C.

TnSeq sequencing library preparation

Genomic DNA was extracted from two library aliquots using a GeneJet Genomic DNA Purification Kit (ThermoScientific). Initially, three separate PCR amplification reactions were performed using the JM1 forward primer and one of the JM2, JM3 or JM4 reverse primers (Table S5), which arbitrarily bind on the *Caulobacter* genome. Subsequently, the three reaction mixtures were combined to serve as the template for a PCR amplification with primers JM7 and JM28 (Table S5) to amplify the transposon junctions and append unique molecular identifies (UMIs). Next, unwanted DNA fragments (such as primers) were selected against by using AMPure XP beads (Beckman Coulter) according to the manufacturer's instructions. Illumina indexes were added using primers JM8-JM27 (Table S5), followed by another cleanup step using AMPure XP beads. The resulting indexed library was purified on a 1% agarose gel using a GeneJet Gel Extraction Kit (Thermo Fisher Scientific) and sequenced on a NextSeq 2000 (llumina) according to the manufacturer's instructions.

Competitive transposon mutant fitness assays

Eight frozen aliquots of the randomly barcoded *C. crescentus* mutagenesis library were pooled and resuspended in 100 ml M2G and recovered for 13 hours at 30˚C to achieve a mid-exponential phase culture (OD_{600nm} = 0.4). 3 ml of the culture was collected by centrifugation (2 min, 17000 x *g*) and the pellet was stored at -20°C to serve as t=0 in the Tn-Seq analysis. The remaining culture was diluted with M2G to an OD_{600nm} of 0.08 and separated out into 16 cultures of 25 ml (i.e. four replicates for each of the four conditions (M2G, C-starvation, N-starvation and P-starvation). Next, the cultures were centrifuged (5 min, 7197 x *g*) twice and resuspended in 25 ml of the final medium to be incubated at 30˚C. After eight hours of incubation, the M2G-cultures were back-diluted 1:10 to a final concentration of 25 ml to preserve mid-exponential growth. After another eight hours of growth, 3 ml of each of the M2G-cultures was collected by centrifugation (2 min, 17000 x *g*) and the pellets were stored at -20°C. After 24 hours of incubation, 3 ml of the C-, N- and P-starvation cultures was collected by centrifugation (2 min, 17000 x *g*) and the pellets were stored at -20°C. 4 ml of the same cultures was transferred to 76 ml of M2G-medium for 16 hours of incubation at 30°C, after which 3 ml of the cultures was collected by centrifugation (2 min, 17000 x *g*) and the pellets were stored at -20°C.

gDNA was extracted from the frozen pellets using a GeneJet Genomic DNA Purification Kit (Thermo Fisher Scientific). The transposon barcodes were amplified and prepared for Illumina sequencing by PCR amplification using one of the forward indexing primers (JM40-68) and a pool of five phased reverse primers (JM30-34). The resulting reaction mixtures were purified on a 1% agarose gel using a GeneJet Gel Extraction Kit (Thermo Fisher Scientific) and sequencing was performed on a NextSeq 2000 (Illumina) according to the manufacturer's instructions.

RB-TnSeq data analysis

To map transposon insertion sites with transposon barcodes for the *C. crescentus* Tn5 transposon mutagenesis library, reads covering both locations were processed using TnSeq-pipe (available at https://github.com/m-jahn/TnSeq-pipe) and using model_pKMW7_KJ as model for the read structure (2). This pipeline maps transposon insertions sites to the *C. crescentus* genome (genome assembly ASM2200v1) and summarizes the read counts per barcode. The TnSeq-pipe.Rmd script from TnSeq-pipe was adapted for this work and is made available at https://github.com/julienmortier/RB-TnSeq_starvation. Barcodes associated with multiple transposon insertion sites or barcodes not mapping to a single insertion sites with at least two reads were filtered out of the analysis.

The samples taken during the competitive fitness assay were analysed with ReBar (available at https://github.com/m-jahn/rebar) (2), which calculates fitness scores for each barcode and for each gene based on the method of Wetmore *et al.*, 2015. The barcode counts from the M2G exponential phase culture at t_0 were used as the reference group. Next, the Barseq_analysis.R script was used to further analyse the fitness scores (available at https://github.com/julienmortier/RB-TnSeq_starvation). All barcodes in open reading frames not coding for proteins were removed from the analysis and the average fitness score for each gene was calculated across all replicates for each condition (**Data S3**). A one-sample Student's t-test was then performed to assess whether the mean fitness score significantly differed from zero.

LIVE/DEAD staining

Viability of P-starved cells was analyzed with the LIVE/DEAD BacLight fluorescence stain from Life Technologies (Thermo Fisher Scientific), as previously described (3). The two dye components were combined at a 2:1 ratio of component A with component B. A 1 µl volume of the mixed dye was added to 300 µl of liquid aliquots from P-starved cultures, and the mixture was incubated for 30 min at room temperature (RT) in the dark. Phase-contrast and fluorescence images were taken using a T_i eclipse inverted research microscope (Nikon) with a 100×/1.45 numerical aperture (NA) and a 100x objective (Nikon). Fiji (ImageJ) was used for image processing.

Spot assays with *pitA* expression strains

To test the sensitivity of the *ppk* mutant strains to expression of *E. coli pitA* in phosphate replete conditions, three independent clones of each strain (WT, Δ*ppk1*, Δ*ppk2* and Δ*ppk1*Δ*ppk2*) in which *pitA* from *E. coli* was integrated into the chromosome under the control of the xylose inducible promoter P_{xylX} (P_{xylX}::*pitA*^{Ec}) were inoculated in 2 ml PYE growth medium containing spectinomycin (Spec) and streptomycin (Strep) into sterile 12-well transparent plates with flat bottom and lid, and incubated at 30° C with orbital shaking at 180 rpm. Overnight cultures were then diluted 1:20 into 2 ml nutrient-replete M2G containing Spec/Strep in new 12-well plates and incubated at 30° C with orbital shaking at 180 rpm. Then overnight cultures were diluted and standardised to OD_{600nm} 0.05 in fresh nutrient-replete M2G or M2GX (M2G

containing 0.3% xylose) in new 12-well plates and incubated at 30° C with orbital shaking at 180 rpm. Cultures were serially diluted prior incubation (T0) and after 6 hours of growth (T6h), and 5µl spotted onto M2G agar plates containing Spec/Strep. Spots were left to dry before plates were inverted and incubated at 30°C for 48 hours. Plates were imaged using the Amersham Imager 600 (GE Healthcare).

Supplementary Figures

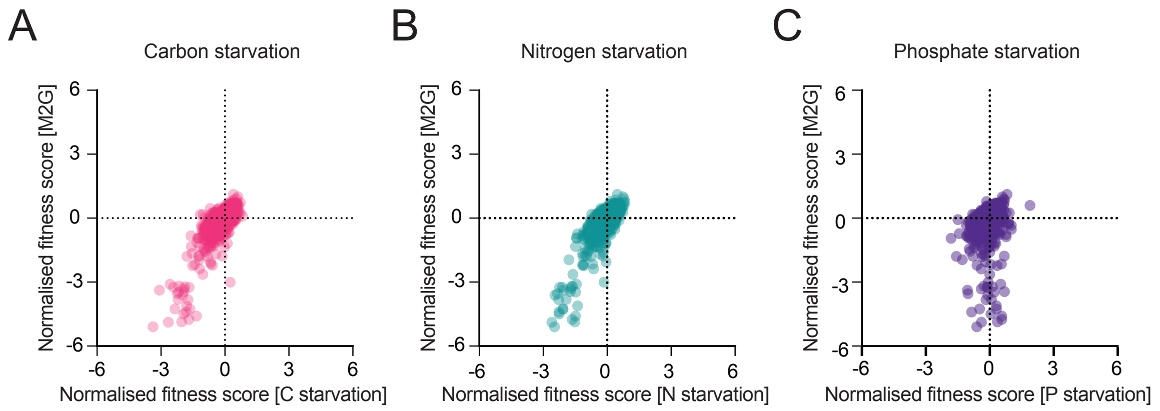

Supplementary Figure S1: RB-TnSeq data showing the effects of carbon (A), nitrogen (B) and phosphate (C) starvation. Normalised fitness scores are plotted for the three starvation conditions against the fitness scores determined for growth in M2G.

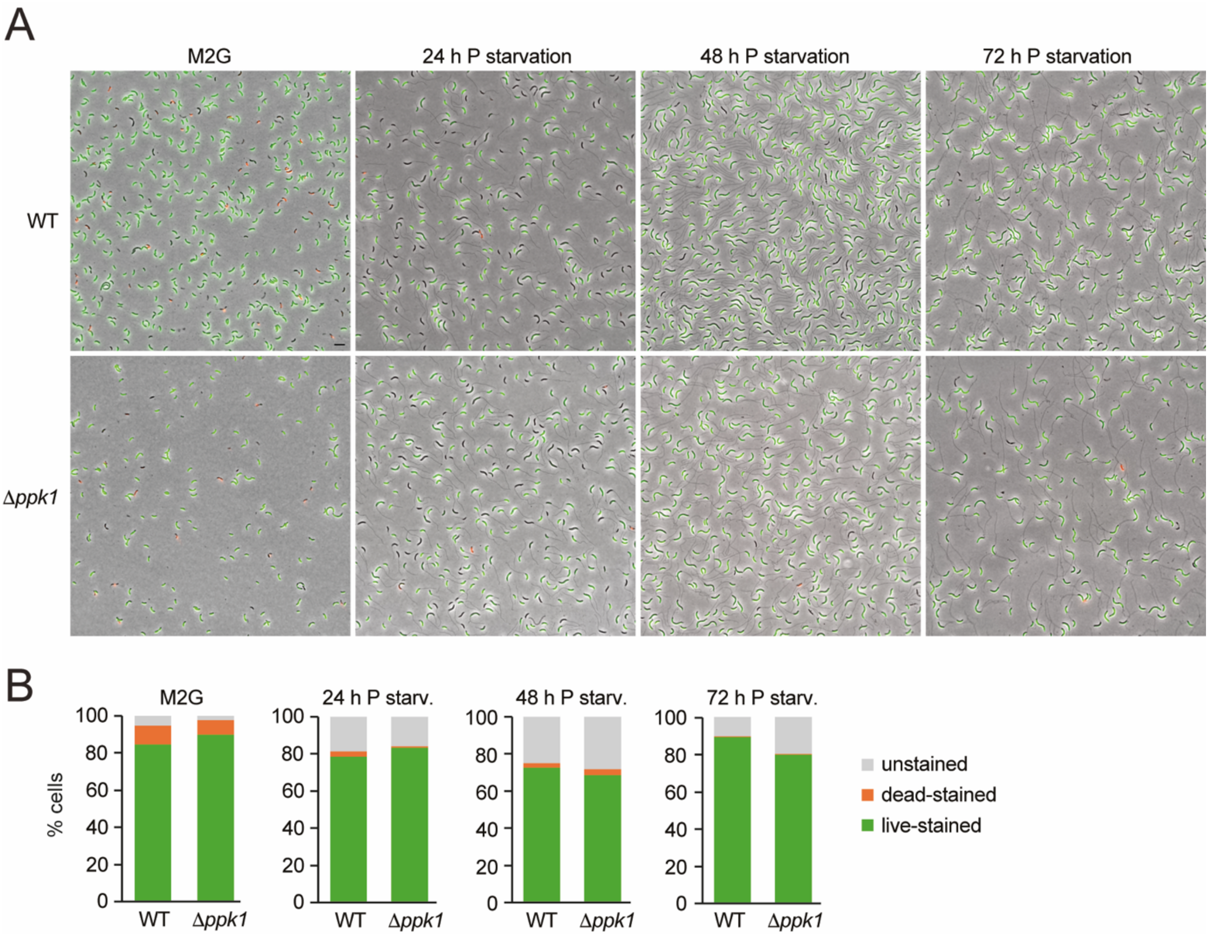

Supplementary Figure S2: LIVE/DEAD staining indicates that the wild type and the Δ*ppk1* mutant do not lose cell integrity during P starvation. (**A**) Representative images of the wild type and the Δ*ppk1* mutant after staining with the LIVE/DEAD stain. Phase-contrast and fluorescence images were overlayed. Cells with a compromised membrane that are considered to be dead or dying stain red, whereas cells with an intact membrane stain green. Scale bar is 5 µm. (**B**) Quantification of fractions of dead-stained (orange), alive-stained (green) and unstained cells (grey) of the wild type and the Δ*ppk1* mutant in M2G and after 24 h, 48 h and 72 h of P starvation. The data represent averages of at least two independent experiments, in which between 288 and 542 P-starved cells were analysed. For the M2G condition, data are representative of two independent experiments and based on the analysis of 177 and 202 cells of the wild type and the Δ*ppk1* mutant, respectively.

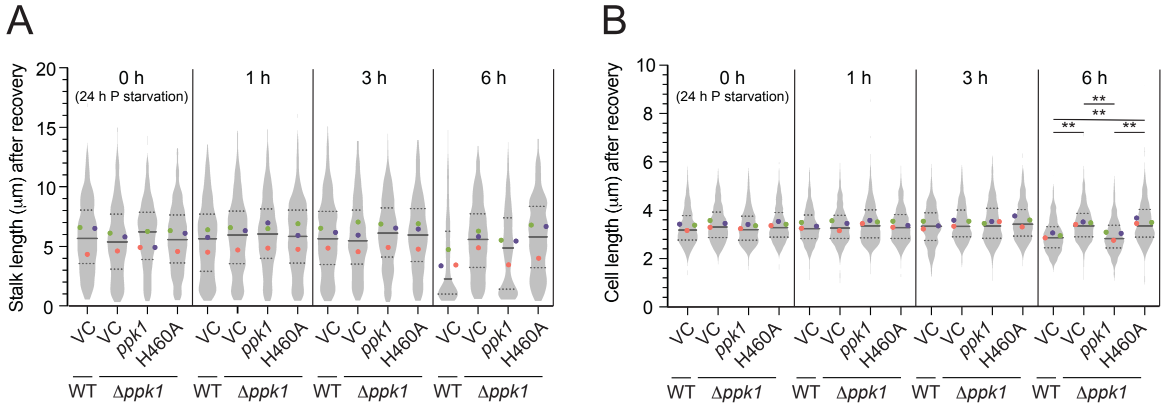

Supplementary Figure S3: Additional stalk length and cell length data for the wild type, the Δ*ppk1* mutant and the *ppk1* and *ppk1*^{H460A} complemented strains. (**A**) Violin plot of stalk lengths for the wild type with the vector control (VC) and the Δ*ppk1* mutant containing either VC or either the *ppk1*- or the *ppk1*^{H460A}-expressing plasmid, after 0, 1, 3 and 6 h recovery. T=0 h corresponds to the time point when phosphate starved cells were diluted 1:10 into nutrient replete M2X medium. Grey shaded area is representative of 375 random stalk lengths from three biological replicates. Dotted lines represent inter-quartile range, dashed lines represent median stalk length. The average of each replicate is indicated by colored circles, with error bars showing the standard deviation of the averages. (**B**) Violin plot of cell lengths from microscopy for the same condition as shown in **A**. 675 cell lengths from 3 biological replicates represented as described above. Asterisks denote statistical significance for each pairwise comparison. Significance determined by a One-Way ANOVA with Bonferroni correction; ** $P<0.01$

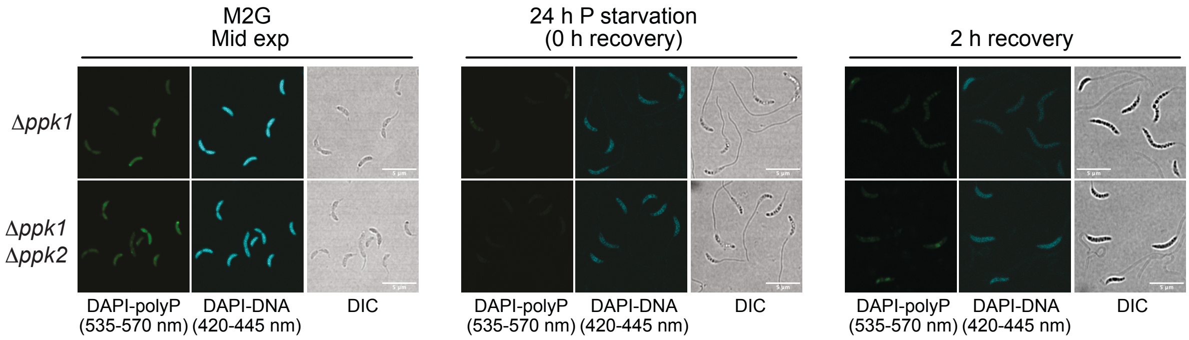

Supplementary Figure S4: Fluorescence microscopy following DAPI-staining indicates that the Δ*ppk1* and Δ*ppk1* Δ*ppk2* mutants do not contain intensively stained polyP granules. DAPI-staining and imaging of cells was done before starvation (M2G), after P starvation (24 h) and recovery (2 h). DAPI-stained polyP was visualised using emission wavelengths of 535–570 nm, while DAPI-stained DNA was visualised at 420–445 nm for comparison. DIC images are shown to visualise the cells.

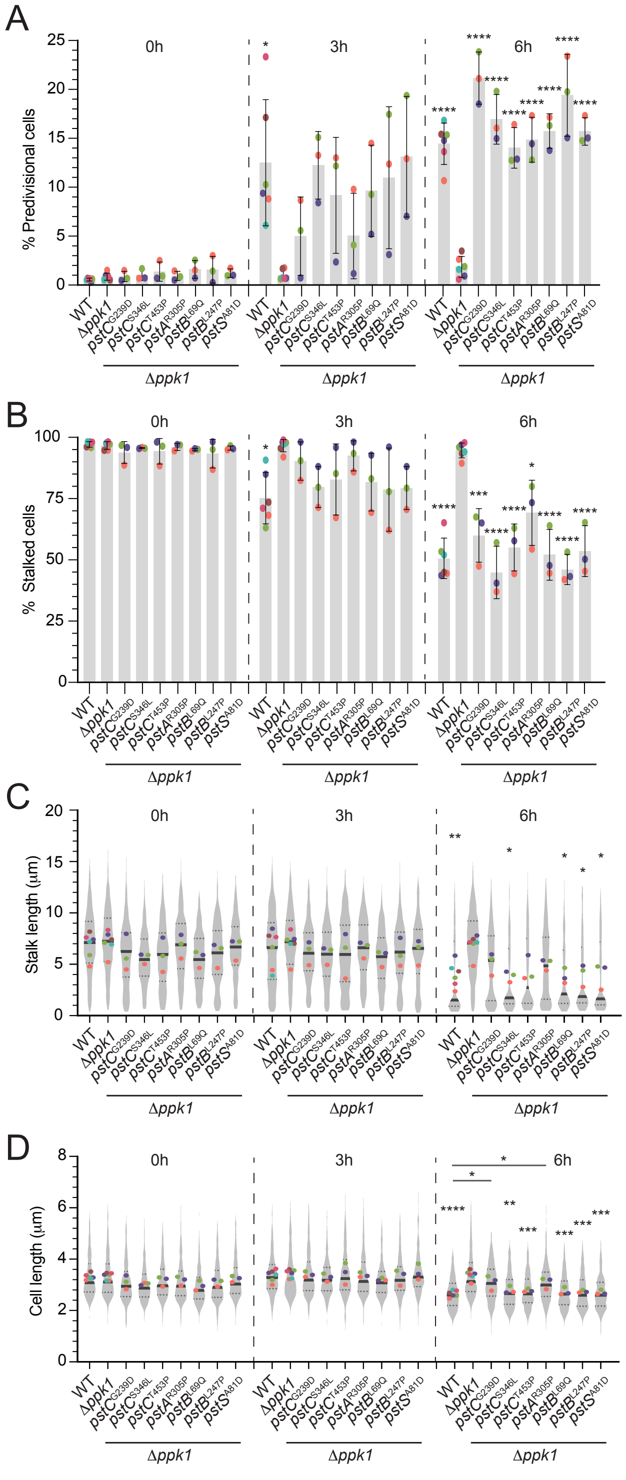

Supplementary Figure S5: Additional phenotypic data of the suppressor mutants bypassing the defects of the Δ*ppk1* mutant. (**A**) Proportion of predivisional cells and non-predivisional stalked cells (**B**) of the wild type, the Δ*ppk1* mutant and the suppressor strains after 0, 3, 6 h of recovery. T=0 h corresponds to the time point when phosphate starved cells (for 24 h) were transferred to phosphate replete M2G medium. Asterisks denote statistical significance between the indicated strain and the Δ*ppk1* mutant unless otherwise indicated with a black bar. (**C**) Violin plot of stalk lengths of the wild type, the Δ*ppk1* mutant and the suppressor strains after 0, 3, 6 h of recovery. Grey shaded area is representative of 291 random stalk lengths from three biological replicates. Dotted lines represent inter-quartile range, dashed

lines represents median stalk length. The average of each replicate is indicated by colored circles. (**D**) Violin plot of cell lengths of the strains and time points as in **C**. Grey shaded area is representative of 684 random cell lengths from three biological replicates. Significance was determined for all panels by a One-Way ANOVA with Bonferroni correction; * $P<0.05$, ** $P<0.01$, *** $P<0.001$, **** $P<0.0001$.

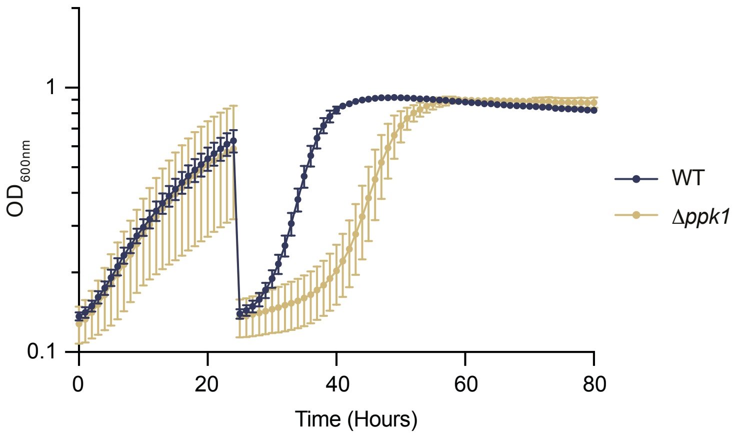

Supplementary Figure S6: The Δ*ppk1* mutant is able to regrow after P starvation when PYE is used as the recovery medium. Growth curves of wildtype (WT) *C. crescentus* and the Δ*ppk1* mutant during P starvation and subsequent recovery in PYE medium. After 24 h of P starvation cultures were diluted 1:10 into fresh PYE and grown for a further 56 hours. Results are displayed as averages from two biological replicates over nine technical replicates and are representative of two independent experiments.

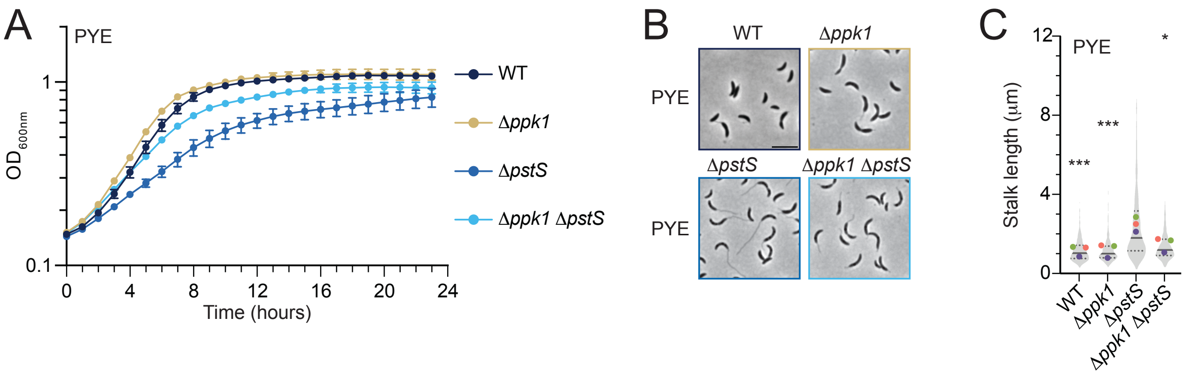

Supplementary Figure S7: The Δ*ppk1* mutation alleviates the phenotypes of a Δ*pstS* mutant in PYE medium. (**A**) Growth curve of WT *C. crescentus* and the Δ*ppk1*, Δ*pstS* and Δ*ppk1*Δ*pstS* mutants in PYE. Cultures were standardised and grown for 24 hours with OD_{600nm} readings taken every hour. Three independent experiments each in technical triplicate were carried out and the average of technical replicates plotted. (**B**) Phase contrast micrographs of the strains shown in **A** grown in PYE medium. Cells grown in the exponential phase were imaged live. Scale bar is 5µm. Micrographs representative of three independent experiments. (**C**) Violin plot of stalk lengths for microscopy in panel B. Grey shaded area represents 672 random stalk lengths from three biological replicates for cells grown in PYE. Dotted lines represent inter-quartile range, dashed lines represent median stalk length. The average of each replicate is indicated by coloured circles. Asterisks denote statistical significance between indicated strain and the Δ*pstS* mutant. Significance determined by a One-Way ANOVA with Bonferroni correction; * $P<0.05$, *** $P<0.001$.

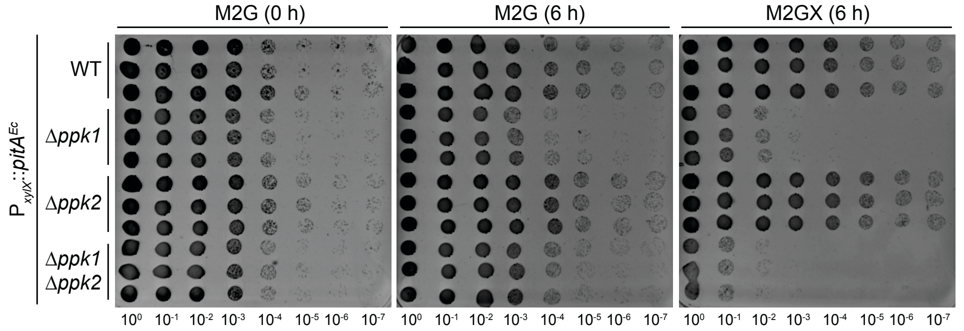

Supplementary Figure S8: Δ*ppk1* mutants do not tolerate heterologous expression of *pitA*, encoding the P_i transporter of *E. coli*. Viability assays of WT *C. crescentus* and the Δ*ppk1*, Δ*ppk2* and Δ*ppk1*Δ*ppk2* mutants, in which *pitA* from *E. coli* was integrated into the chromosome under the control of the xylose inducible promoter P_{xylX} (P_{xylX}::*pitA*^{Ec}). Overnight cultures grown in M2G Spec/Strep were diluted to OD660~0.05 and either immediately spotted on M2G Spec/Strep plates (0 h) or grown for additional 6 hours in either liquid M2G medium or xylose-containing M2GX medium (6 h) before being spotted on M2G Spec/Strep plates. Three biological replicates are shown for each strain per condition.

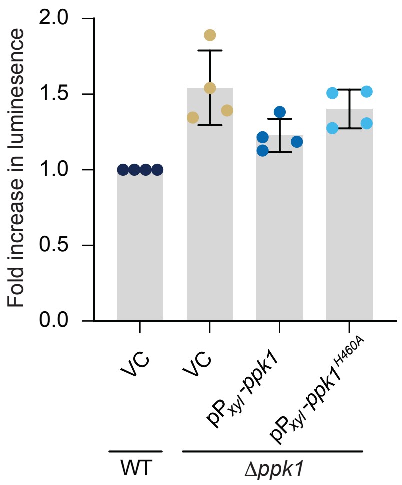

Supplementary Figure S9: ATP levels in the wild type, the Δ*ppk1* mutant and the complemented strains when grown in M2G medium without prior starvation. BacTiter-Glo assay of wildtype *C. crescentus* harbouring the vector control (VC) and the Δ*ppk1* mutant, either with VC or plasmids containing *ppk1* or *ppk1*^{H460A} under the control of a xylose inducible promoter (P_{xyl}). Samples were taken from cultures grown exponentially in M2GX.

Supplementary Tables

Strain	Genotype	Construction	Reference
NA1000	WT (CB15N)		(4)
RH2350 (KJ1085)	Δ*ppk1*	Integration of pHR913 into NA1000 + sucrose selection	This study
KJ1250	WT + pRXMCS2 (vector control)	Transformation of pRXMCS2 into NA1000	This study
KJ1248	Δ*ppk1* + pRXMCS2 (vector control)	Transformation of pRXMCS2 into RH2350	This study
KJ1249	Δ*ppk1* + pRXMCS2-*ppk1* (pP_{xyl}-*ppk1*)	Transformation of pMW8 into RH2350	This study
KJ1272	Δ*ppk1* + pRXMCS2-$ppk1^{H460A}$ (pP_{xyl}-$ppk1^{H460A}$)	Transformation of pMW13 into RH2350	This study
RH2351 (KJ1086)	Δ*ppk2*	Integration of pHR914 into NA1000 + sucrose selection	This study
RH2352 (KJ1087)	Δ*ppk1* Δ*ppk2*	Integration of pHR913 into RH2351 + sucrose selection	This study
FC1580 (KJ1165)	Δ*ppx1* *xylX*::pMT585		(5)
KJ1253	Δ*ppk1* Δ*ppx1*	Integration of FC681into RH2350	This study
KJ1260	Δ*ppk1* $pstS^{A81D}$	Spontaneous suppressor mutation in RH2350 background	This study
KJ1289	Δ*ppk1* $pstC^{G239D}$	Spontaneous suppressor mutation in RH2350 background	This study
KJ1290	Δ*ppk1* $pstC^{S346L}$	Spontaneous suppressor mutation in RH2350 background	This study
KJ1291	Δ*ppk1* $pstC^{T435P}$	Spontaneous suppressor mutation in RH2350 background	This study
KJ1292	Δ*ppk1* $pstA^{R305P}$	Spontaneous suppressor mutation in RH2350 background	This study
KJ1293	Δ*ppk1* $pstB^{L69Q}$	Spontaneous suppressor mutation in RH2350 background	This study
KJ1294	Δ*ppk1* $pstB^{L247P}$	Spontaneous suppressor mutation in RH2350 background	This study
JK158 (KJ1220)	*pstS*::$spec^R$		(6)
KJ1288	Δ*ppk1* *pstS*::$spec^R$	Integration of pJK25 into RH2350	This study
JK2 (KJ1219)	Δ*phoB*		(6)
KJ1299	Δ*ppk1* Δ*phoB*	Integration of pJK3 into RH2350	This study

Table S1. Strains used in this study. List of strains used in this study and their method of construction.

Name	Description	Reference
pRXMCS2	Low copy number vector for expression in *C. crescentus* under control of xylose	(7)
pNTPS138	*sacB*-containing suicide plasmid used for double homologous recombination, *kanR*	M.R. Alley
pXMCS-1	Integrative vector for expression in *C. crescentus* under control of xylose, $spec^R/strep^R$	(7)

Table S2. General plasmids used in this study. List of plasmids used to generate plasmids listed in Table S3.

Name	Description	Construction
pMW8	pRXMCS2-*ppk1* (pP$_{xyl}$-*ppk1*)	*ppk1* was amplified with oMW39/40, gel extracted. Gibson assembly was used to insert into the backbone which was digested with EcoRI/NheI.
pMW13	pRXMCS2-$ppk1^{H460A}$ (pP$_{xyl}$-$ppk1^{H460A}$)	*ppk1* was amplified in two fragments to introduce a H460A mutation. Primer pairs oMW45/oMW53 and oMW53/oMW40 were used for PCR. The resulting fragments were integrated into pRXMCS2 backbone digested with EcoRI/NheI using Gibson assembly.
FC681	pNPTS138-Δ*ppx1*	Gifted by Sean Crosson (5)
pJK25	pNPTS138-*pstS::*$spec^R$	Gifted by Martin Thanbichler (6)
pKJ3	pNTPS138-Δ*phoB*	Gifted by Martin Thanbichler (6)
pHR913	pNPTS138-Δ*ppk1*	Upstream and downstream regions of *C. crescentus ppk1*(*CCNA_01782*) were amplified from NA1000 by PCR with primers 1072/1073 and 1074/1075, respectively. The PCR products were then digested with Bam HI/Eco RI and Eco RI/Hind III, respectively; and ligated into the pNPTS138 vector cut with Hind III and Bam HI.
pHR914	pNPTS138-Δ*ppk2*	Upstream and downstream regions of *C. crescentus ppk2* (*CCNA_03529*) were amplified from NA1000 by PCR with primers 1120/1121 and 1122/1123, respectively. The PCR products were then digested with Bam HI/Eco RI and Eco RI/Hind III, respectively; and ligated into the pNPTS138 vector cut with Hind III and Bam HI.
pHR883	pXC-1	*E. coli pitA* was amplified from MG1655 gDNA by PCR with primers 1982/1983. The PCR was then digested with *Sal* I/*Xba* I; and ligated into the pBXMCS-2 vector cut with *Sal* I/*Xba* I.
pHR1589	pXC-1-$pitA^{Ec}$	*E. coli pitA* was amplified from MG1655 gDNA by PCR with primers 2070/2071. The PCR was then digested with *Pac* I/*Sac* I; and ligated into the pXC-1 vector cut with *Pac* I/*Sac* I.

Table S3. Plasmids constructed or used for this study. List of plasmids used in this study and details of their construction.

Name	Sequence (5'→3')
oMW43	tacttgccgtccccacatgtta
oMW44	ggagaaaataccgcatcaggcgccat
oMW39	agttttggggagacgaccatat**gaattc**atgaccgacgccctgccgat
oMW40	atcccccgggctgca**gctagc**tcaccgcttgcgcttgggcccca
oMW45	agacgaccatat**gaattc**atgaccgacgccctgccgat
oMW46	cgggctgca**gctagc**tcaccgcttgcgcttgggc
oMW53	gtcgactggaagaccGCcgccaagctgtc
oMW54	gacagcttggcgGCggtcttccagtcgac
oMW76	gttggaggaaggttgcgcgt
oMW77	tggcctacggcacctgctac
oMW70	aagttgtctggacgatggcgt
oMW71	aggaatgggtcgagcttcgt
1072	tgcttagtc**aagctt**gtcggtcgccgaaatcgc
1073	tgcttagtc**gaattc**ctctccccttgtgggagaagg
1074	tgcttagtc**gaattc**cgccaacctcaatgacgaggc
1075	tgcttagtc**ggatcc**tgggcggttttctccacacc
1120	tc**aagctt**gccccagcttgacgaaatcg
1121	tcgttgcagctggac**gaattc**gg
1122	tc**gaattc**atcctgcggtgatcttcccg
1123	tc**ggatcc**actatctggccagactgccc
2070	ggatccccgggta**ttaattaa**gcgaggaaacgCATatgctacatttgtttgctgg
2071	cctaagtaactaa**gagctc**ttacaggaactgcaaggaga

Table S4. Primers used for strain constructions in this study. List of primers used for the construction of strains in this study. Base pairs in green overlap with the targeted plasmid, in bold the restriction sites and in red overlap with the gene to be integrated into the plasmid.

Name	Sequence (5'→3')
JM1	CGCCCTGCAGGGATGTCCACGAG
JM2	GTCTCGTGGGCTCGGAGATGTGTATAAGAGACAGNNNNNNNNNNACGCC
JM3	GTCTCGTGGGCTCGGAGATGTGTATAAGAGACAGNNNNNNNNNNCCTGG
JM4	GTCTCGTGGGCTCGGAGATGTGTATAAGAGACAGNNNNNNNNNNCCTCG
JM7	GTCTCGTGGGCTCGGAGATG
JM28	TCGTCGGCAGCGTCAGATGTGTATAAGAGACAGNNNNGGATGTCCACGAGGTCTCT
JM8	CAAGCAGAAGACGGCATACGAGATTCGCCTTAGTCTCGTGGGCTCGG
JM9	CAAGCAGAAGACGGCATACGAGATCTAGTACGGTCTCGTGGGCTCGG
JM10	CAAGCAGAAGACGGCATACGAGATTTCTGCCTGTCTCGTGGGCTCGG
JM11	CAAGCAGAAGACGGCATACGAGATGCTCAGGAGTCTCGTGGGCTCGG
JM12	CAAGCAGAAGACGGCATACGAGATAGGAGTCCGTCTCGTGGGCTCGG
JM13	CAAGCAGAAGACGGCATACGAGATCATGCCTAGTCTCGTGGGCTCGG
JM14	CAAGCAGAAGACGGCATACGAGATGTAGAGAGGTCTCGTGGGCTCGG
JM15	CAAGCAGAAGACGGCATACGAGATCCTCTCTGGTCTCGTGGGCTCGG
JM16	CAAGCAGAAGACGGCATACGAGATAGCGTAGCGTCTCGTGGGCTCGG
JM17	CAAGCAGAAGACGGCATACGAGATCAGCCTCGGTCTCGTGGGCTCGG
JM18	AATGATACGGCGACCACCGAGATCTACACTAGATCGCTCGTCGGCAGCGTC
JM19	AATGATACGGCGACCACCGAGATCTACACCTCTCTATTCGTCGGCAGCGTC
JM20	AATGATACGGCGACCACCGAGATCTACACTATCCTCTTCGTCGGCAGCGTC
JM21	AATGATACGGCGACCACCGAGATCTACACAGAGTAGATCGTCGGCAGCGTC
JM22	AATGATACGGCGACCACCGAGATCTACACGTAAGGAGTCGTCGGCAGCGTC
JM23	AATGATACGGCGACCACCGAGATCTACACACTGCATATCGTCGGCAGCGTC
JM24	AATGATACGGCGACCACCGAGATCTACACAAGGAGTATCGTCGGCAGCGTC
JM25	AATGATACGGCGACCACCGAGATCTACACCTAAGCCTTCGTCGGCAGCGTC
JM26	AATGATACGGCGACCACCGAGATCTACACCGTCTAATTCGTCGGCAGCGTC
JM27	AATGATACGGCGACCACCGAGATCTACACTCTCTCCGTCGTCGGCAGCGTC
JM30	AATGATACGGCGACCACCGAGATCTACACTATAGCCTACACTCTTTCCCTACACGACGC TCTTCCGATCNNNNNNGTCGACCTGCAGCGTACG
JM31	AATGATACGGCGACCACCGAGATCTACACTATAGCCTACACTCTTTCCCTACACGACGC TCTTCCGATCNNNNNGTCGACCTGCAGCGTACG
JM32	AATGATACGGCGACCACCGAGATCTACACTATAGCCTACACTCTTTCCCTACACGACGC TCTTCCGATCNNNNGTCGACCTGCAGCGTACG
JM33	AATGATACGGCGACCACCGAGATCTACACTATAGCCTACACTCTTTCCCTACACGACGC TCTTCCGATCNNNGTCGACCTGCAGCGTACG
JM34	AATGATACGGCGACCACCGAGATCTACACTATAGCCTACACTCTTTCCCTACACGACGC TCTTCCGATCNNGTCGACCTGCAGCGTACG
JM40	CAAGCAGAAGACGGCATACGAGATCGTGATGTGACTGGAGTTCAGACGTGTGCTCTTCC GATCTGATGTCCACGAGGTCTCT
JM41	CAAGCAGAAGACGGCATACGAGATACATCGGTGACTGGAGTTCAGACGTGTGCTCTTCC GATCTGATGTCCACGAGGTCTCT
JM42	CAAGCAGAAGACGGCATACGAGATGCCTAAGTGACTGGAGTTCAGACGTGTGCTCTTCC GATCTGATGTCCACGAGGTCTCT
JM43	CAAGCAGAAGACGGCATACGAGATTGGTCAGTGACTGGAGTTCAGACGTGTGCTCTTCC GATCTGATGTCCACGAGGTCTCT
JM44	CAAGCAGAAGACGGCATACGAGATCACTGTGTGACTGGAGTTCAGACGTGTGCTCTTCC GATCTGATGTCCACGAGGTCTCT
JM45	CAAGCAGAAGACGGCATACGAGATATTGGCGTGACTGGAGTTCAGACGTGTGCTCTTCC GATCTGATGTCCACGAGGTCTCT

JM46	CAAGCAGAAGACGGCATACGAGATGATCTGGTGACTGGAGTTCAGACGTGTGCTCTTCCGATCTGATGTCCACGAGGTCTCT
JM47	CAAGCAGAAGACGGCATACGAGATTCAAGTGTGACTGGAGTTCAGACGTGTGCTCTTCCGATCTGATGTCCACGAGGTCTCT
JM48	CAAGCAGAAGACGGCATACGAGATCTGATCGTGACTGGAGTTCAGACGTGTGCTCTTCCGATCTGATGTCCACGAGGTCTCT
JM49	CAAGCAGAAGACGGCATACGAGATAAGCTAGTGACTGGAGTTCAGACGTGTGCTCTTCCGATCTGATGTCCACGAGGTCTCT
JM50	CAAGCAGAAGACGGCATACGAGATGTAGCCGTGACTGGAGTTCAGACGTGTGCTCTTCCGATCTGATGTCCACGAGGTCTCT
JM51	CAAGCAGAAGACGGCATACGAGATTACAAGGTGACTGGAGTTCAGACGTGTGCTCTTCCGATCTGATGTCCACGAGGTCTCT
JM52	CAAGCAGAAGACGGCATACGAGATTTGACTGTGACTGGAGTTCAGACGTGTGCTCTTCCGATCTGATGTCCACGAGGTCTCT
JM53	CAAGCAGAAGACGGCATACGAGATGGAACTGTGACTGGAGTTCAGACGTGTGCTCTTCCGATCTGATGTCCACGAGGTCTCT
JM54	CAAGCAGAAGACGGCATACGAGATTGACATGTGACTGGAGTTCAGACGTGTGCTCTTCCGATCTGATGTCCACGAGGTCTCT
JM55	CAAGCAGAAGACGGCATACGAGATGGACGGGTGACTGGAGTTCAGACGTGTGCTCTTCCGATCTGATGTCCACGAGGTCTCT
JM56	CAAGCAGAAGACGGCATACGAGATCTCTACGTGACTGGAGTTCAGACGTGTGCTCTTCCGATCTGATGTCCACGAGGTCTCT
JM57	CAAGCAGAAGACGGCATACGAGATGCGGACGTGACTGGAGTTCAGACGTGTGCTCTTCCGATCTGATGTCCACGAGGTCTCT
JM58	CAAGCAGAAGACGGCATACGAGATTTTCACGTGACTGGAGTTCAGACGTGTGCTCTTCCGATCTGATGTCCACGAGGTCTCT
JM59	CAAGCAGAAGACGGCATACGAGATGGCCACGTGACTGGAGTTCAGACGTGTGCTCTTCCGATCTGATGTCCACGAGGTCTCT
JM60	CAAGCAGAAGACGGCATACGAGATCGAAACGTGACTGGAGTTCAGACGTGTGCTCTTCCGATCTGATGTCCACGAGGTCTCT
JM61	CAAGCAGAAGACGGCATACGAGATCGTACGGTGACTGGAGTTCAGACGTGTGCTCTTCCGATCTGATGTCCACGAGGTCTCT
JM62	CAAGCAGAAGACGGCATACGAGATCCACTCGTGACTGGAGTTCAGACGTGTGCTCTTCCGATCTGATGTCCACGAGGTCTCT
JM63	CAAGCAGAAGACGGCATACGAGATGCTACCGTGACTGGAGTTCAGACGTGTGCTCTTCCGATCTGATGTCCACGAGGTCTCT
JM64	CAAGCAGAAGACGGCATACGAGATATCAGTGTGACTGGAGTTCAGACGTGTGCTCTTCCGATCTGATGTCCACGAGGTCTCT
JM65	CAAGCAGAAGACGGCATACGAGATGCTCATGTGACTGGAGTTCAGACGTGTGCTCTTCCGATCTGATGTCCACGAGGTCTCT
JM66	CAAGCAGAAGACGGCATACGAGATAGGAATGTGACTGGAGTTCAGACGTGTGCTCTTCCGATCTGATGTCCACGAGGTCTCT
JM67	CAAGCAGAAGACGGCATACGAGATCTTTTGGTGACTGGAGTTCAGACGTGTGCTCTTCCGATCTGATGTCCACGAGGTCTCT
JM68	CAAGCAGAAGACGGCATACGAGATTAGTTGGTGACTGGAGTTCAGACGTGTGCTCTTCCGATCTGATGTCCACGAGGTCTCT

Table S5. Primers used for TnSeq experiment.

Legends for Datasets

Data S1: Fitness scores per transposon insertion site (i.e. transposon barcode) that occurs in a protein-encoding gene as determined by the ReBar pipeline using the method described in Wetmore *et al.* (1) for the P-, N- and C-starvation and recovery conditions and the M2G control. Additionally, this table includes further details for each transposon barcode such as the genomic insertion site, barcode sequence, count data, etc. Additional information with respect to gene and protein names, Gene Ontology descriptions, function, etc. was added from the UniProt database.

Data S2: Fitness scores per protein-encoding gene as determined by the ReBar pipeline using the method described in Wetmore *et al.* (1) for the P-, N- and C-starvation and recovery conditions and the M2G control. Additional information with respect to gene and protein names, Gene Ontology descriptions, function, etc. was added from the UniProt database.

Data S3: ReBar fitness scores from Supplementary information S2 averaged over all replicates per treatment. This data corresponds to the data represented in Fig. 1B-D and Fig. S1. Additional information with respect to gene and protein names, Gene Ontology descriptions, function, etc. was added from the UniProt database.

SI References

1. K. M. Wetmore *et al.*, Rapid quantification of mutant fitness in diverse bacteria by sequencing randomly bar-coded transposons. *mBio* **6**, e00306-00315 (2015).
2. M. Jahn *et al.*, Protein allocation and utilization in the versatile chemolithoautotroph *Cupriavidus necator*. *Elife* **10** (2021).
3. K. Heinrich, D. J. Leslie, M. Morlock, S. Bertilsson, K. Jonas, Molecular Basis and Ecological Relevance of *Caulobacter* Cell Filamentation in Freshwater Habitats. *mBio* **10** (2019).
4. M. Evinger, N. Agabian, Envelope-associated nucleoid from *Caulobacter crescentus* stalked and swarmer cells. *J Bacteriol* **132**, 294-301 (1977).
5. C. C. Boutte, J. T. Henry, S. Crosson, ppGpp and polyphosphate modulate cell cycle progression in *Caulobacter crescentus*. *J Bacteriol* **194**, 28-35 (2012).
6. M. Billini, T. Hoffmann, J. Kühn, E. Bremer, M. Thanbichler, The cytoplasmic phosphate level has a central regulatory role in the phosphate starvation response of *Caulobacter crescentus*. *Communications Biology* **7** (2024).
7. M. Thanbichler, A. A. Iniesta, L. Shapiro, A comprehensive set of plasmids for vanillate- and xylose-inducible gene expression in *Caulobacter crescentus*. *Nucleic Acids Research* **35**, e137-e137 (2007).
